# KMT2D acetylation by CREBBP reveals a cooperative functional interaction at enhancers in normal and malignant germinal center B cells

**DOI:** 10.1073/pnas.2218330120

**Published:** 2023-03-09

**Authors:** Sofija Vlasevska, Laura Garcia-Ibanez, Romain Duval, Antony B. Holmes, Rahat Jahan, Bowen Cai, Andrew Kim, Tongwei Mo, Katia Basso, Rajesh K. Soni, Govind Bhagat, Riccardo Dalla-Favera, Laura Pasqualucci

**Affiliations:** ^a^Institute for Cancer Genetics, Columbia University, New York, NY 10032; ^b^Department of Pathology and Cell Biology, Columbia University, New York, NY 10032; ^c^Proteomics and Macromolecular Crystallography Shared Resource, Columbia University, New York, NY 10032; ^d^Herbert Irving Comprehensive Cancer Center, Columbia University, New York, NY 10032; ^e^Department of Genetics and Development, Columbia University, New York, NY 10032; ^f^Department of Microbiology and Immunology, Columbia University, New York, NY 10032

**Keywords:** B cell lymphoma, KMT2D, acetylation, germinal center, CREBBP

## Abstract

Heterozygous inactivating mutations of the KMT2D methyltransferase and the CREBBP acetyltransferase are among the most common genetic alterations in B cell lymphoma and co-occur in 40 to 60% of follicular lymphoma (FL) and 30% of EZB/C3 diffuse large B cell lymphoma (DLBCL) cases, suggesting they may be coselected. Here, we show that combined germinal center (GC)–specific haploinsufficiency of *Crebbp* and *Kmt2d* synergizes in vivo to promote the expansion of abnormally polarized GCs, a common preneoplastic event. These enzymes form a biochemical complex on select enhancers/superenhancers that are critical for the delivery of immune signals in the GC light zone and are only corrupted upon dual *Crebbp*/*Kmt2d* loss, both in mouse GC B cells and in human DLBCL. Moreover, CREBBP directly acetylates KMT2D in GC-derived B cells, and, consistently, its inactivation by FL/DLBCL-associated mutations abrogates its ability to catalyze KMT2D acetylation. Genetic and pharmacologic loss of CREBBP and the consequent decrease in KMT2D acetylation lead to reduced levels of H3K4me1, supporting a role for this posttranslational modification in modulating KMT2D activity. Our data identify a direct biochemical and functional interaction between CREBBP and KMT2D in the GC, with implications for their role as tumor suppressors in FL/DLBCL and for the development of precision medicine approaches targeting enhancer defects induced by their combined loss.

## Significance

Our findings provide a mechanistic explanation to the frequent coselection of inactivating CREBBP and KMT2D mutations in lymphoma. They reveal a biochemical and functional cooperativity at select enhancer networks whose disruption facilitates the preneoplastic expansion of germinal center B cells. The identification of KMT2D as a direct target of CREBBP acetylation has implications for the design of strategies targeting tumors where these genes are concurrently mutated.

The two most common forms of lymphoma in adults, diffuse large B cell lymphoma (DLBCL) and follicular lymphoma (FL), originate from the malignant transformation of antigen-experienced germinal center (GC) B cells (1, 2). Although significant progress has been made in the diagnosis and treatment of these cancers, including the recent success of immune and chimeric antigen receptor (CAR) T cell products, nearly one third of DLBCL patients do not respond or fail to achieve durable complete remission (3), and FL remains an essentially incurable disease (4). Furthermore, as many as 3% of FL cases transform into a fatal, high-grade malignancy each year, typically in the form of DLBCL (4).

A genetic hallmark of FL and DLBCL is the presence of somatic mutations in histone/chromatin modifier genes collectively found in over 90% of FL cases and ~60% of DLBCL cases (5–7). Among these lesions, the most common are inactivating mutations and/or deletions of the genes encoding the KMT2D histone lysine methyltransferase and the CREBBP acetyltransferase, accounting for 80 to 60% of FL cases and 60 to 40% of DLBCL cases belonging to the recently identified EZB/C3 genetic cluster (5–10). Mutations of *CREBBP* and *KMT2D* largely occur in heterozygosis and are among the earliest events in the evolutionary history of FL, being introduced in a common ancestral clone that subsequently progresses to FL or transformed FL (tFL) through divergent evolution, following the acquisition of additional genetic alterations (10–13). In vivo, individual deletion of *Crebbp* or *Kmt2d* in the GC was insufficient alone to drive full malignant transformation but accelerated the development of FL/DLBCL-like lymphomas in a Bcl2-transgenic background, consistent with a haploinsufficient tumor suppressor role (14–17).

CREBBP and KMT2D are the major regulators of H3K27/H3K18 acetylation (Ac) and H3K4 monomethylation (H3K4me1), respectively (18, 19). In GC B cells, they exert this function at selected enhancers/superenhancers (E/SEs) that are activated in the GC light zone (LZ) upon specific signals involved in cell fate decisions and the commitment to terminal differentiation (14–17). In addition, CREBBP counteracts the activity of the BCL6 transcriptional repressor both directly, through acetylation-mediated impairment of its protein function (17, 20), and indirectly, by depositing histone activation marks on the regulatory domains of virtually all BCL6 target genes (14, 17), thus priming them for reexpression upon GC exit. Furthermore, acetylation by CREBBP or by its paralog p300 is an essential requirement for the activation of the p53 tumor suppressor (21), which is also a transcriptional target of BCL6 in the GC (22). Mutational inactivation of CREBBP and KMT2D is thus thought to contribute to lymphoma development by decommissioning enhancer networks that are required for post-GC differentiation while tipping the balance between the oncogenic function of BCL6 at the expense of p53.

Notably, loss-of-function mutations of *CREBBP* and *KMT2D* co-occur in ~50% of FLs and ~30% of EZB/C3 DLBCLs (8–10, 12, 13), suggesting they may be coselected in lymphomagenesis on the basis of some functional interaction. Further supporting this hypothesis is the notion that epigenetic mechanisms entail the integrated activity of multiple macromolecular complexes providing different layers of mutual information, and that the function of chromatin modifiers is influenced by the local chromatin environment, including its histone modification pattern (23). In this context, recent studies in differentiating adipocytes have shown that, to fully activate its target genes, CREBBP requires the presence of KMT2D, which primes E/SEs of cell identity genes through the deposition of H3K4me1 marks (24). Moreover, a feedforward regulatory loop has been described between Kmt2d and p300 in mouse embryonic stem cells (ESCs) to establish active enhancer landscapes (25).

Based on these observations, we set out to examine the impact of combined *CREBBP* and *KMT2D* haploinsufficiency in vivo and explored how these two tumor suppressors functionally interact in normal and malignant GC B cells to promote oncogenesis. We uncovered a synergistic interplay whereby heterozygous loss of *Crebbp* and *Kmt2d* promotes the preneoplastic expansion of abnormal GCs (the target structure of FL and DLBCL development) by perturbing a unique set of enhancers that are distinct from those affected by either mutation alone and are involved in the regulation of the B:T cell immune synapse and in memory B cell identity. Moreover, we identified the KMT2D protein as a physiological direct target of CREBBP-mediated acetylation, providing evidence for the impact of this posttranslational modification in modulating KMT2D function.

## Results

### Combined Haploinsufficiency of *Kmt2d* and *Crebbp* Promotes Abnormal GC Expansion In Vivo.

To examine the combined role of *Kmt2d* and *Crebbp* haploinsufficiency in the GC, we generated compound *Cγ1^Cre/+^* mouse models where these two genes can be conditionally deleted (individually or in combination) in the GC B cell population following immunization with T cell–dependent antigens (26). We then used multicolor flow cytometry and immunohistochemistry (IHC) of splenic B cells to analyze the GC response 10 d after intraperitoneal injection of sheep red blood cells (SRBCs), when this reaction reaches its peak.

**Fig. 1.**
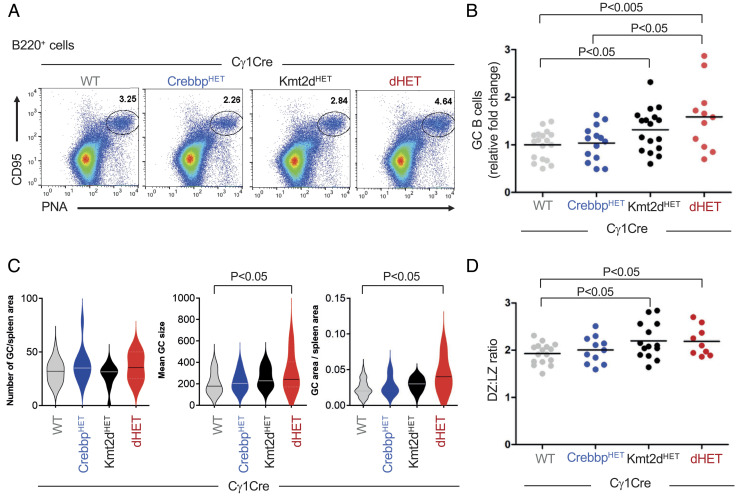
Haploinsufficiency of *Crebbp* and *Kmt2d* synergizes to promote the expansion of GC B cells. (*A*) Representative flow cytometric analysis of splenic B220^+^ cells from *Kmt2d^+/+^Crebbp^+/+^ Cγ1^Cre/+^*(WT), *Crebbp^fl/+^Cγ1^Cre/+^*(*Crebbp^HET^*), *Kmt2d^fl/+^Cγ1^Cre/+^*(*Kmt2d^HET^*), and *Kmt2d^fl/+^Crebbp^fl/+^Cγ1^Cre/+^* (dHET) mice analyzed 10 d after SRBC immunization. GC B cells are identified as CD95^+^PNA^hi^ cells, and numbers in each panel indicate the percentage in the gate. (*B*) Percentage of GC B cells in mice from the indicated genotypes analyzed 10 d after SRBC immunization. Data are from five independent experiments and are expressed as relative changes compared to the mean percentage in WT littermates from the same experiment, arbitrarily set as 1 (n = 11 to 18 mice/genotype; see also *SI Appendix*, Fig. S1). One-way ANOVA with Bonferroni post hoc correction. (*C*) Mean GC number, GC size, and overall GC area (per spleen section) in mice of the indicated genotypes, measured in pixels using ImageJ (n = 2 independent experiments with 3 to 4 mice/genotype and 3 sections/mouse). Student’s *t* test. (*D*) GC DZ:LZ ratio in the same mouse cohorts (n = 9 to 16 mice/genotype from four independent experiments). Student’s *t* test. In all plots, only statistically significant *P* values are indicated.

While no significant differences were observed between *Crebbp^fl/+^Cγ1^Cre/+^* (*Crebbp^HET^*)and *Crebbp^+/+^Kmt2d^+/+^Cγ1^Cre/+^*wild-type (WT) littermates, as reported, and the expected mild increase in GC B cells was seen in *Kmt2d^fl/+^Cγ1^Cre/+^*(*Kmt2d^HET^*) mice (16, 17), heterozygous loss of both genes in *Crebbp^fl/+^Kmt2d^fl/+^Cγ1^Cre/+^* (dHET) mice led to a significant expansion of the GC population, with a mean 1.5 fold increase over WT (*P *< 0.05, two-tailed Student’s *t* test) (Fig. 1 *A* and *B* and *SI Appendix*, Fig. S1 *A*–*C*). This finding was not due to a higher number of GCs (Fig. 1*C*, *Left*) but rather to the formation of significantly larger GC structures, as documented by IHC of the specific markers PNA and BCL6 (Fig. 1*C*, *Middle* and *Right* and *SI Appendix*, Fig. S1*D*). Analysis of the dark zone (DZ) and LZ B cell subsets, representing the two main functional compartments of the GC (27), revealed a modest but statistically significant alteration in GC polarization, exemplified by a higher DZ:LZ ratio (Fig. 1*D* and *SI Appendix*, Fig. S1*E*) and reflecting a more pronounced increase in the number of DZ B cells (*SI Appendix*, Fig. S1*F*). Interestingly, although no major differences in affinity maturation were detected across the four genotypes 12 d upon immunization with NP–KLH, based on analysis of the hotspot W33R mutation that confers increased affinity for NP to the rearranged immunoglobulin V186.2 gene (28), the proportion of highly mutated clones (>4 nucleotide changes) was significantly higher in dHET mice compared to both single-mutant and WT littermates (*SI Appendix*, Fig. S1*G*; *P *< 0.05, two-tailed Student’s *t* test). This finding is compatible with the hypothesis that B cells with reduced dosage of *Crebbp* and *Kmt2d* may undergo prolonged exposure to the somatic hypermutation machinery in the context of an altered GC dynamics. Thus, haploinsufficiency of *Crebbp* and *Kmt2d* cooperates in promoting the expansion of abnormal GCs, recapitulating a preneoplastic phenotype that typically precedes overt tumor formation in most genetically engineered mouse models of human lymphomas (29).

### CREBBP and KMT2D Coexist on a Shared Set of E/SEs Linked to Immune Signaling Genes.

**Fig. 2.**
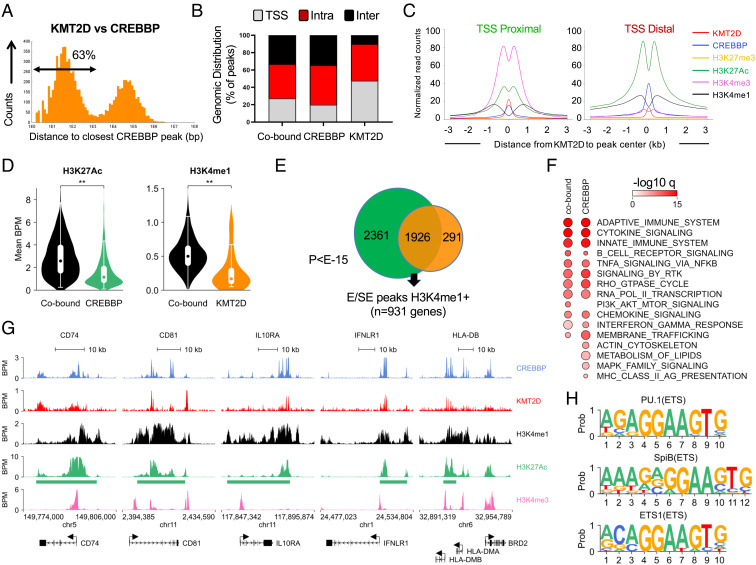
CREBBP and KMT2D occupy shared and distinct E/SEs linked to immune signaling genes. (*A*) Distance plots of KMT2D ChIP-seq peaks in human GC B cells, as related to the closest CREBBP peak. (*B*) Distribution pattern of KMT2D and CREBBP mapping in human GC B cells. Chromatin domains bound by KMT2D-only, CREBBP-only, and both proteins (co-bound) are defined as TSS-proximal (–2/+1 kb), intragenic (i.e., regions within a gene), and intergenic (i.e., regions between annotated genes). (*C*) Histone modification pattern at CREBBP/KMT2D-cobound regions shown separately for TSS-proximal and TSS-distal chromatin domains. Signal is centered on the KMT2D peak. (*D*) Violin plots of H3K27Ac and H3K4me1 enrichment in regions cooccupied by CREBBP and KMT2D as compared to regions bound by these enzymes individually (**P < 0.01; Mann–Whitney test). (*E*) Venn diagram of shared and unique regions bound by KMT2D and CREBBP at E/SEs in GC B cells. (*F*) Top positively enriched (*q *< 0.05 after Benjamini–Hochberg correction) biological programs/signaling pathways in the gene lists identified in (*E*). Analyses were performed using the top 500 genes (based on ChIPseeker *P* value) and signatures from the MSigDB Hallmark and CP/C2 gene sets (KEGG and REACTOME). The size of the circle is proportional to the number of genes in the overlap (see Dataset S2 for the complete list). (*G*) ChIP-seq tracks of CREBBP, KMT2D, H3K4me1, H3K27Ac, and H3K4me3 at representative chromatin domains bound by KMT2D and/or CREBBP. Green bars below the H3K27Ac track denote domains identified as SEs by ROSE. Genes included in the region are aligned below the tracks (only the main transcript is shown, with arrows indicating the transcription start site). Genomic coordinates based on hg19. (*H*) Top significantly enriched TF binding motifs identified in CREBBP/KMT2D-cobound regions.

To gain insights into the mechanism by which loss of CREBBP and KMT2D functionally interact, we first looked at their relative occupancy on chromatin by interrogating ChIP-seq data of KMT2D and CREBBP mapping obtained from human purified GC B cells (n = 2 independent pools/antibody) (16, 17). We found that over 60% of the KMT2D-bound regions (85% if restricting the analysis to E/SEs) overlap or are in close proximity (<1 kb) to a CREBBP-bound chromatin domain (Fig. 2*A*). Cooccupied regions were mostly represented by promoter-distal intergenic or intragenic sequences (Fig. 2*B*) displaying features of active E/SEs (H3K27Ac^+^H3K4me1^+^H3K4me3^-^) (Fig. 2*C*; see also Dataset S1) and included virtually all GC-specific SEs predicted by the ROSE algorithm (30). Interestingly, the enrichment in H3K27Ac was significantly higher in chromatin domains occupied by both CREBBP and KMT2D as compared to CREBBP-only regions, and the same was true for H3K4me1 when compared to regions occupied by KMT2D alone (Fig. 2*D*), suggesting these enzymes may influence each other’s activity.

Gene set enrichment analysis (GSEA) of the top 500 genes linked to cobound, H3K4me1^+^ E/SEs (*Methods*) revealed a significant overrepresentation of programs related to BCR signaling, cytokines and chemokines stimulation, G-protein signaling, and transcription factors of relevance to the GC reaction (e.g. IRF4 and BCL6), consistent with a shared role for KMT2D and CREBBP in the modulation of these biological functions (Fig. 2 *E*–*G* and Dataset S2). In line with these data, the top significantly enriched transcription factor binding motifs in the “cobound” chromatin domains were those recognized by ETS family members, including PU1, SPIB, and ETS1 (Fig. 2*H* and *SI Appendix*, Fig. S2 *A* and *B*). These proteins are known to regulate BCR-mediated signal transduction, as well as signaling emanating from the CD40 receptor, the BAFFR, and the Toll-like receptors (31, 32), and are thus necessary for the appropriate response of B cells to environmental cues. Additionally, a few programs were preferentially enriched in CREBBP-only-bound domains, including MHC-II antigen presentation molecules (Fig. 2 *F* and *G*). FACS analysis of surface MHC-II, a known CREBBP target (13), confirmed its reduced expression in mouse *Crebbp^HET^* and dHET, but not in *Kmt2d^HET^* GC B cells, indicating these enzymes also have nonredundant functions (*SI Appendix*, Fig. S2*C*).

### Dual Loss of Crebbp and *Kmt2d* Is Required to Disrupt a Select LZ-Associated Program That Can Be Tracked in Human DLBCL.

**Fig. 3.**
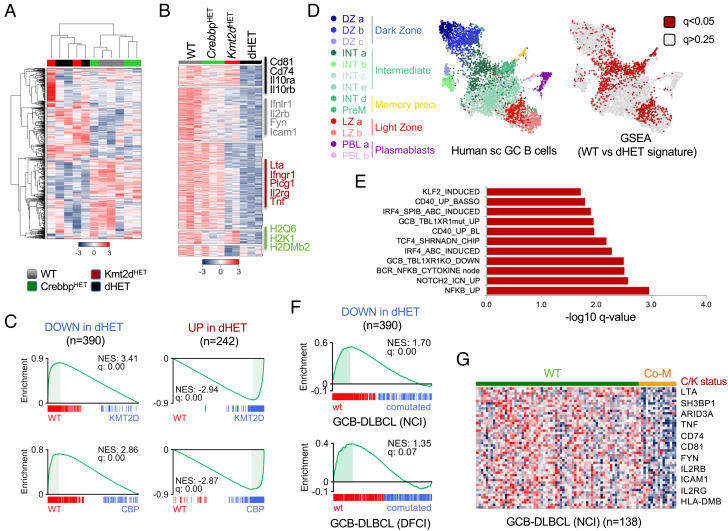
Dual loss of CREBBP and KMT2D perturbs a select LZ-associated program in normal GCs and DLBCL. (*A*) Unsupervised hierarchical clustering of RNA-seq profiles from purified mouse GC B cells of the indicated genotypes. Color scale represents the z score. (*B*) Heat map of significantly down-regulated transcripts in either *Kmt2d*^HET^, *Crebbp*^HET^, or dHET GC B cells as compared to WT (*q* < 0.05 after Benjamini–Hochberg correction). Scale bar indicates the z score (see also Dataset S3). (*C*) GSEA enrichment plots of genes significantly down-regulated (*Left*) or up-regulated (*Right*) in dHET mice across the rank of differentially expressed genes in WT vs *Kmt2d*^HET^ or WT vs Crebbp^HET^ GC B cells. (*D*) GSEA of GC single cell signatures in WT vs dHET GC B cells. On the left, UMAP projection of scRNA profiles obtained from human GC B cells color-coded according to 13 distinct B cell differentiation states identified in ref 33 (see original Fig. 2*A*). On the right, the enrichment q values obtained by GSEA of the 13 sc-associated signatures in WT vs dHET mice are overlaid onto the same UMAP plot. Red color identifies signatures significantly down-regulated in dHET cells (q < 0.05) as a read-out for the depletion of specific subpopulations. Gray color identified nonsignificant changes (>0.25), and the full details of the analysis are provided in Dataset S4. (*E*) Top significantly dysregulated molecular programs in dHET GC B cells, as identified by GSEA using the Signature DB datasets (see also Dataset S4 and *SI Appendix*, Fig. S3 *B* and *C*). (*F*) GSEA enrichment plots of dHET down-regulated genes in GCB-DLBCL (CREBBP/KMT2D comutated vs WT). See also *SI Appendix*, Fig. S3 *D* and *E*. (*G*) Heat map of 70 leading-edge genes identified in (*F*) (DLBCL-NCI cohort). Representative genes are indicated. C/K, *CREBBP/KMT2D* genetic status; co-M, comutated (tumors carrying mutations in CREBBP-only and KMT2D-only not included).

To define specific and shared functions of CREBBP and KMT2D in the GC, we performed RNA-seq analysis of sorted Cd95^+^Pna^+^ B cells from mice with single or double heterozygous loss of these two genes. Unsupervised hierarchical clustering separated WT and *Crebbp*^HET^ cells from *Kmt2d*^HET^ and dHET cells, indicating *Kmt2d* loss has a stronger impact on this population compared to *Crebbp* loss (Fig. 3*A*). However, the changes imposed by *Crebbp/Kmt2d* codeletion extended far beyond what would be predicted based on individual gene loss, comprising 390 transcripts that were significantly down-regulated and 243 that were up-regulated (DESeq2, FDR < 0.05; FC ≥ 1.2) (Fig. 3*B* and *SI Appendix*, Fig. S3*A*, and Dataset S3). Importantly, 76% of the down-regulated transcripts (295/390) were exclusively affected in dHET cells (nominal q value <0.05), whereas only 24% were also reduced in *Kmt2d*^HET^ (93/390) or *Crebbp*^HET^ cells (2/390), with a small set of genes showing antagonistic responses between the two genotypes (Fig. 3*B*). Nonetheless, a gradient of expression indicative of intermediate phenotypes could be appreciated in the single mutants for one third of these transcripts (second group from the top in Fig. 3*B*). GSEA using the dHET signature (i.e., the list of transcripts differentially expressed between WT and dHET cells) confirmed their significant and coordinated enrichment in both *Kmt2d*^HET^ and *Crebbp*^HET^ mice (Fig. 3*C*). We conclude that combined deletion of *Kmt2d* and *Crebbp* imposes both qualitative and quantitative (additive) changes, reflecting the rewiring of a select Crebbp/Kmt2d-dependent “enhanceosome” distinct from that affected by either lesion alone.

We next interrogated the transcriptomic profile of dHET GC B cells for enrichment in specific biological programs by using GSEA of a curated list of gene sets from the MSig Database (DB), Signature DB (https://lymphochip.nih.gov/signaturedb/), and 13 GC B cell states that we recently defined based on single-cell transcriptomic analysis of human cells (33). We found a significant depletion of transcripts associated with LZ B cells and intermediate B cell subsets recirculating between the LZ and DZ (Fig. 3*D* and *SI Appendix*, Fig. S3*B*). These include genes implicated in the CD40 (e.g., Nfkb1a and Nfkb1e) and IFN signaling pathways (e.g., Ifngr, Ifnlr1, and Il10Rb), TNF receptor pathway (e.g., Lta, Tnf, and several receptors of the TNF superfamily), interleukin responses (Il2rg and IL2rb), BCR engagement (Cd81, Fyn), and antigen presentation (HLA class I and II, Cd74, Tapbp), as well as integrins (Icam1, Itgb2) (*SI Appendix*, Fig. S3*C* and Fig. 3*E*). Thus, a prominent consequence of dual *Crebbp/Kmt2d* haploinsufficiency is the impaired expression of molecules that are critical for the communication between B cells and T cells or stromal cells typically occurring in the GC LZ (27, 34, 35), which may explain in part the altered polarity observed in the dHET GCs. The prememory B cell signature was also negatively enriched in dHET GCs (e.g., Rasgrp2 and Tnfrsf13b) (Fig. 3*D* and *SI Appendix*, Fig. S3*B*), although selected genes appeared to be up-regulated (e.g., Cd44, Bank1, and Cd47), suggesting desynchronization of this transcriptional program. The expression of several transcripts linked to protection from apoptosis (Bcl2l1 and Cd44) or the control of G1–S cell cycle progression (Cdk6) was increased in dHET cells and may contribute to GC expansion (Dataset S3).

Cross-species comparison of the mouse data with human DLBCL transcriptomic profiles showed that the “dHET down-regulated signature” was significantly depleted in tumors carrying concurrent *CREBBP/KMT2D* mutations but less so in single *CREBBP-* or *KMT2D-*mutant cases, as documented by GSEA of two independent DLBCL datasets (overall, 617 samples, of which 192 GCB-DLBCL) (Fig. 3 *F* and *G* and *SI Appendix*, Fig. S3 *D*–*F*). Enrichment was specifically observed within the GCB subtype of DLBCL, where *CREBBP* and *KMT2D* mutations typically segregate, confirming the relevance of these findings to the human tumors.

Collectively, these data demonstrate that CREBBP and KMT2D orchestrate shared and unique regulatory networks in the GC and reveal a select group of enhancers that are only disrupted upon combined gene loss but not upon single gene depletion both in normal GC B cells and in human DLBCL.

### CREBBP and KMT2D Physically Interact.

**Fig. 4.**
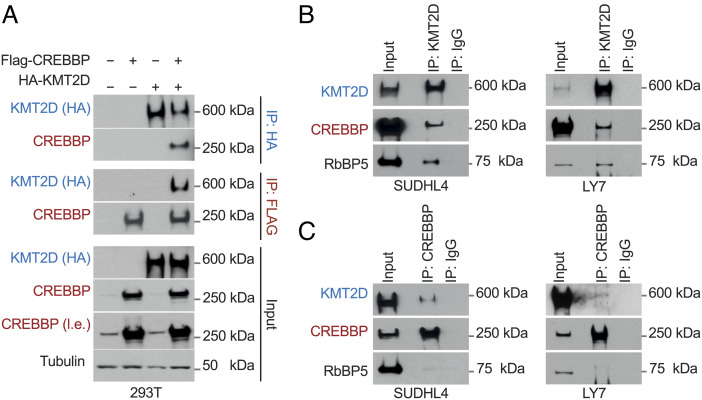
CREBBP and KMT2D form a biochemical complex in B cells. (*A*) Immunoblot analysis of the indicated proteins in whole-cell extracts (input), KMT2D immunoprecipitates (IP:HA), and CREBBP immunoprecipitates (IP:FLAG) obtained from HEK293-T cells cotransfected with HA-KMT2D and FLAG-CREBBP expression vectors. Tubulin is used as the loading control. L.e., long exposure. (*B*) Physical interaction of CREBBP and KMT2D in nuclear extracts from native DLBCL cells documented by immunoblot analysis of KMT2D (or control IgG) immunoprecipitates with the indicated antibodies. The reverse IP confirms this interaction in panel *C*.

The significant cooccupancy of shared chromatin domains prompted us to ask whether CREBBP and KMT2D can physically interact. To this end, we first performed coimmunoprecipitation assays in 293T cells transfected with an HA-tagged KMT2D expression vector in the presence or absence of FLAG-CREBBP. Immunoblot analysis with specific antibodies revealed that CREBBP is efficiently pulled down with KMT2D in the HA immunoprecipitates, and the reverse was true in the reciprocal immunoprecipitation (Fig. 4*A*). This physical interaction was readily detected in native GC-derived cells exemplified by the two GCB-DLBCL cell lines SUDHL4 and LY7 that carry wild-type KMT2D alleles and intact lysine acetyltransferase genes (KAT1-KAT8 families) (Fig. 4 *B* and *C*). The endogenous RbBP5 component of the COMPASS-like complex (36) was also present in the immunoprecipitates, further confirming this finding. Thus, CREBBP and KMT2D form a biochemical complex in GC B cells that is corecruited to a select subset of enhancers/superenhancers implicated in the physiology of the GC reaction.

### KMT2D Is Acetylated in Normal and Transformed GC B Cells.

**Fig. 5.**
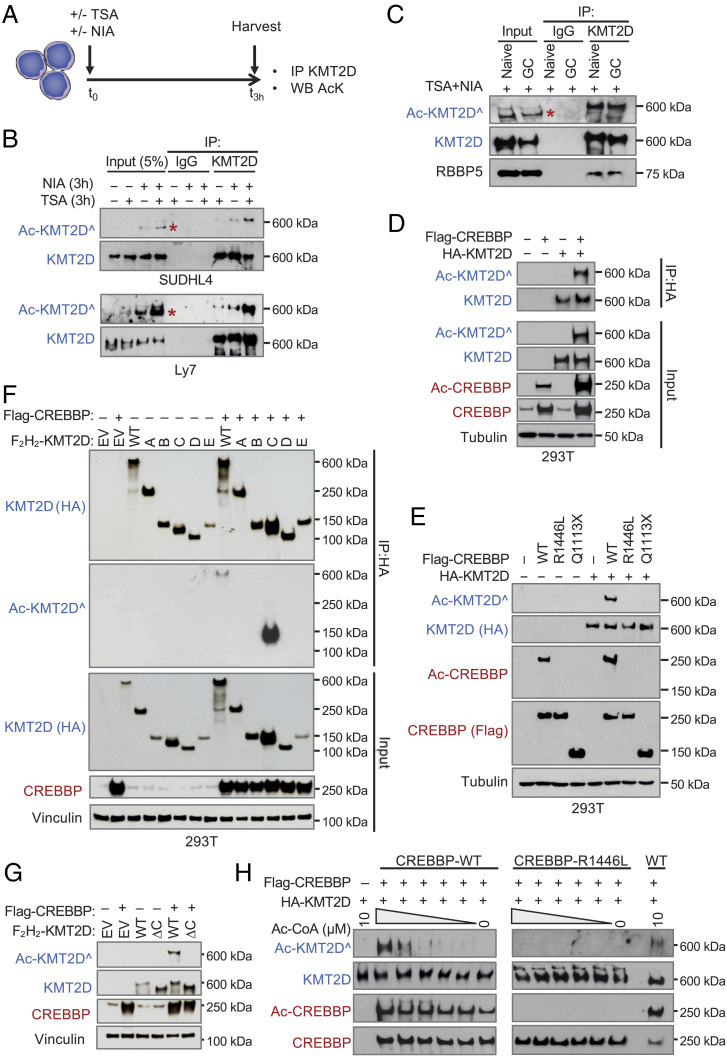
KMT2D is acetylated by CREBBP. (*A*) Experimental plan used for the analysis of KMT2D acetylation in normal B cell subsets and DLBCL cell lines harboring intact *KMT2D* and lysine acetyltransferase (*KAT1* through *KAT8*) genes. (*B*) Immunoblot analysis of the indicated proteins in whole-cell extracts (*Input*) and KMT2D immunoprecipitates (KMT2D-IP) from two DLBCL cell lines treated with TSA and/or NIA. IgG antibodies were used as control for nonspecific binding (CREBBP, p300, and AcCREBBP/p300 levels in the inputs or vinculin loading control in *SI Appendix*, Fig. S4*A*). (*C*) Immunoblot analysis of the indicated proteins in whole-cell extracts (input) and KMT2D immunoprecipitates (KMT2D-IP) from purified naive and GC B cells (*SI Appendix*, Fig. S4 *B* and *C*). In (*B*) and (*C*), Ac-KMT2D was detected using a pan-AcK antibody, and asterisks in the inputs denote a nonspecific band. (*D*) Immunoblot analysis of the indicated proteins in whole-cell extracts (input) or KMT2D immunoprecipitates (IP:HA) from 293T cells cotransfected with vectors expressing HA-KMT2D and FLAG-CREBBP or empty vector as control. The anti-CREBBP antibody detects both endogenous and exogenous CREBBP, and the specific Ac-CBP/p300 antibody documents an active enzyme based on self-acetylation. Inputs are 5% of the lysate used in the IP. (*E*) Immunoblot analysis with anti-AcK antibodies documents the presence of acetylated KMT2D in 293T cells overexpressing CREBBP wild type but not a truncated form lacking the HAT domain (Q1113X) or a point mutant in the HAT domain (R1446L) that is also unable to self-acetylate (6). (*F*) Mapping of the KMT2D acetylated region in transfected 293T cells. Details on the expression constructs used and the list of acetylated lysines validated by mass spectrometry in DLBCL cells are provided in *SI Appendix*, Fig. S5. (*G*) A deletion mutant lacking AA2289-3170 (KMT2D-ΔC) confirms this region is the main target of CREBBP-mediated acetylation. (*H*) In vitro acetyltransferase assay using semipurified HA-KMT2D and FLAG-CREBBP polypeptides (wild type or R1446L mutant) with increasing amounts of Ac-CoA. Immunoblot analysis of AcK reveals a specific, dose-dependent signal upon addition of wild-type CREBBP (*Left*) but not of the enzymatically defective point mutant (*Right*). Documentation of protein purity is provided in *SI Appendix*, Fig. S6.

The observation that KMT2D physically interacts with CREBBP in GC-derived B cells raised the possibility that it could represent a substrate for CREBBP-mediated acetylation, a hypothesis supported by recent proteomic screens reporting KMT2D as part of the “CREBBP/p300” acetylome in mouse embryonic fibroblasts (37). To test this hypothesis, we first examined whether KMT2D is acetylated in the SUDHL4 and Ly7 cell lines by immunoblot analysis of KMT2D immunoprecipitates with antibodies directed against acetylated lysines (AcKs). Cells were cultured for 3 h in the presence or absence of trichostatin A (TSA) and/or niacinamide (NIA), two histone deacetylase inhibitors (HDACi) that are involved in the control of CREBBP/p300-mediated acetylation by dynamically erasing acetylation marks on histone and nonhistone proteins (38) (Fig. 5*A*). As expected, both NIA and to a lesser extent TSA induced a significant increase in CREBBP/p300 self-acetylation, a known regulatory mechanism that stimulates their acetyltransferase activity and binding to proteins (39) (*SI Appendix*, Fig. S4*A*). However, a robust band was detected by the anti-AcK antibody also in the KMT2D immunoprecipitates (Fig. 5*B*), which was maximal upon combined TSA/NIA treatment, indicating that KMT2D is acetylated in these cells and that the deacetylase activity of both HDAC-I/II and sirtuins is involved in the control of this posttranslational modification.

To determine the physiological relevance of these findings, we performed analogous experiments in nontransformed CD77^+^ GC B cells (and as control CD27^−^IgD^+^naive B cells) purified from human tonsils and placed in short-term cultures (2 h) with TSA/NIA (*SI Appendix*, Fig. S4*B*). The expression pattern of the GC master regulator BCL6 (40) and the B cell transcription factor IRF4 (41) verified the identity of the two populations, respectively (*SI Appendix*, Fig. S4*C*), and FACS analysis of cleaved caspase 3 confirmed the lack of detrimental effects on cell viability within this short-time window (*SI Appendix*, Fig. S4*D*). Importantly, both naive and GC B cells showed robust KMT2D acetylation (Fig. 5*C*), confirming the physiological significance of this observation. RbBP5 was efficiently coimmunoprecipitated with KMT2D in all conditions, indicating that acetylated KMT2D participates in COMPASS complex formation (Fig. 5*C*).

We conclude that KMT2D is dynamically acetylated with a very rapid turnover in both normal GC B cells and transformed (KATs/KMT2D unmutated) DLBCL cells.

### CREBBP Directly Acetylates KMT2D.

To explore whether CREBBP is the enzyme catalyzing KMT2D acetylation, we first used transient cotransfection assays followed by immunoprecipitation/western blot in 293T cells. We found that cells cotransfected with KMT2D and CREBBP, but not cells expressing exogenous KMT2D alone, showed a strong acetylation signal corresponding to the 600 kD size predicted for the KMT2D protein (Fig. 5*D*, input). Analysis of the HA immunoprecipitates confirmed that this signal represents acetylated KMT2D polypeptides, documenting that CREBBP is responsible for this posttranslational modification (Fig. 5*D*, IP:HA). In line with this conclusion, KMT2D acetylation was completely abolished when cells were cotransfected with vectors expressing two DLBCL-associated, enzymatically inactive CREBBP proteins, including a C-terminal truncation mutant lacking the HAT domain and a HAT missense mutant (R1446L) we previously demonstrated to be catalytically inactive (6) (Fig. 5*E*). The hyperacetylated domain was mapped to a region in the middle portion of the large KMT2D protein (amino acids 2289-3170), as documented by transient cotransfection assays with 5 FLAG-HA-double-tagged KMT2D deletion mutants tiled across the full-length KMT2D protein (FH-KMT2D A to E), and a full-length mutant lacking specifically this region (FH-KMT2D ΔC) (Fig. 5 *F* and *G* and *SI Appendix*, Fig. S5*A*; the list of acetylated lysines identified by mass spectrometry in SUDHL4 cells stably expressing the KMT2D-C region in *SI Appendix*, Fig. S5 *B*–*D*). In vitro acetylation assays using semipurified KMT2D and CREBBP proteins, together with increasing doses of Ac-CoA, conclusively demonstrated that CREBBP-WT, but not the enzymatically defective R1446L mutant, acetylates KMT2D directly in a dose-dependent manner (Fig. 5*H* and *SI Appendix*, Fig. S6), excluding the potentially confounding activity of other endogenous factors. Thus, the enzymatic function of CREBBP, rather than (or in addition to) its scaffolding function, is required for KMT2D acetylation.

**Fig. 6.**
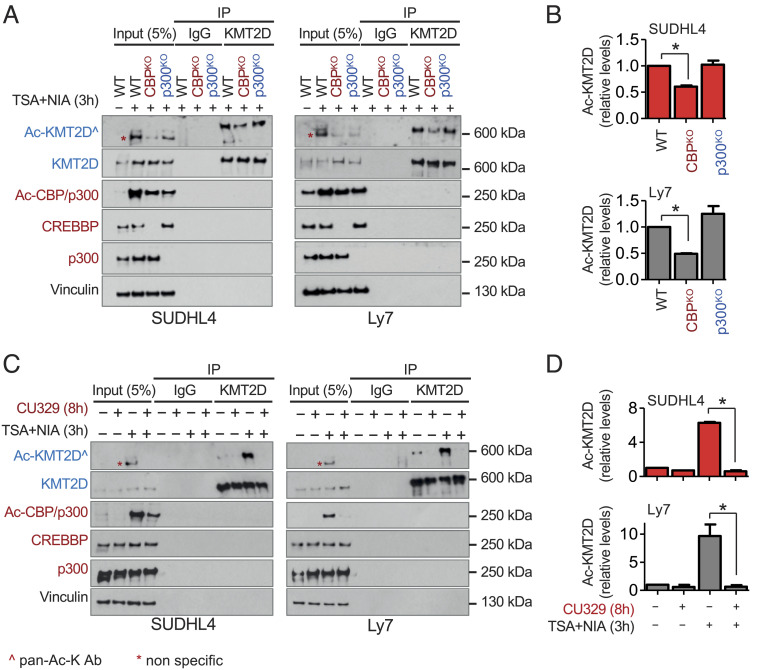
CREBBP is the preferred acetyltransferase for KMT2D. (*A*) Immunoblot analysis of total and Ac-KMT2D in isogenic CREBBP^KO^ and p300^KO^ clones from two DLBCL cell lines as compared to WT control clones obtained by editing a neutral region of the genome (WT). Cells were treated with TSA/NIA for 3 h before IP with a KMT2D antibody or IgG as control. Immunoblot analysis of total CREBBP, p300, and Ac-CBP/p300 controls for efficient knockout of these two acetyltransferases. Vinculin serves as the loading control. (*B*) Quantification of Ac-KMT2D signal in the isogenic clones shown in (*A*), as assessed by densitometry using ImageJ. Data are presented as relative levels compared to the WT control, set as 1, and correspond to two independent experiments (mean ± SD). (*C*) Immunoblot analysis of total and Ac-KMT2D in native SUDHL4 and LY7 cells cultured in the absence (−) or presence (+) of TSA/NIA and a specific CBP/p300 HAT inhibitor prior to IP with KMT2D antibodies. (*D*) Quantification of Ac-KMT2D signal in the same immunoprecipitates, as assessed by densitometry using ImageJ after normalization for total KMT2D levels in the IP (mean ± SD; two independent experiments). In (*A*) and (*C*), Ac-KMT2D was detected by a pan-AcK antibody, and asterisks denote nonspecific signal detected in the inputs. *P < 0.05; Student’s *t* test. Only statistically significant *P* values are indicated in *B* and *D*.

To corroborate these data in native B cells, we engineered SUDHL4 and LY7 to biallelically delete *CREBBP*—or its paralog *EP300*—by using the CRISPR-Cas9 editing tool (n = 2 independent clones each vs 2 control clones edited in a neutral region of the genome) and then measured KMT2D acetylation after a 3-h treatment with TSA/NIA. Interestingly, both isogenic *CREBBP* knockout (KO) cell lines, but not *EP300*^KO^ cells, showed an ~50% reduction in the amount of AcKMT2D compared to wild-type (sgNeutral-transduced) cells (Fig. 6*A*; quantification from three independent experiments in 6*B*). These findings cannot be attributed to differences in total KMT2D protein levels, which were comparable across the different derivative lines, suggesting a preferential requirement for CREBBP over p300 (Fig. 6*A*, IP:KMT2D).

Although measurably reduced, KMT2D acetylation was not completely abrogated in *CREBBP*^KO^ cells, implying that other acetyltransferases could contribute to this modification, in the absence of CREBBP. We hypothesized that the residual signal could be due to the compensatory activity of p300, which we have previously shown to be essential for the fitness of CREBBP-deficient DLBCL cells (42). Because the synthetic lethal interaction between CREBBP and p300 prevents genetic testing via the construction of double-KO cell line models (42), we took advantage of a small-molecule HAT inhibitor (CU329) that is specific for the KAT3 family of acetyltransferases (42, 43) to simultaneously inhibit both enzymes over a short time course. As illustrated in Fig. 6 *C* and *D*, CU329 efficiently blunted KMT2D acetylation in both SUDHL4 and LY7. We conclude that, in GC-derived B cells, CREBBP is the major KMT2D acetyltransferase, with the p300 paralog partially compensating for its loss.

### Tumor-Associated CREBBP HAT Missense Mutations Abrogate Its Ability to Acetylate KMT2D.

**Fig. 7.**
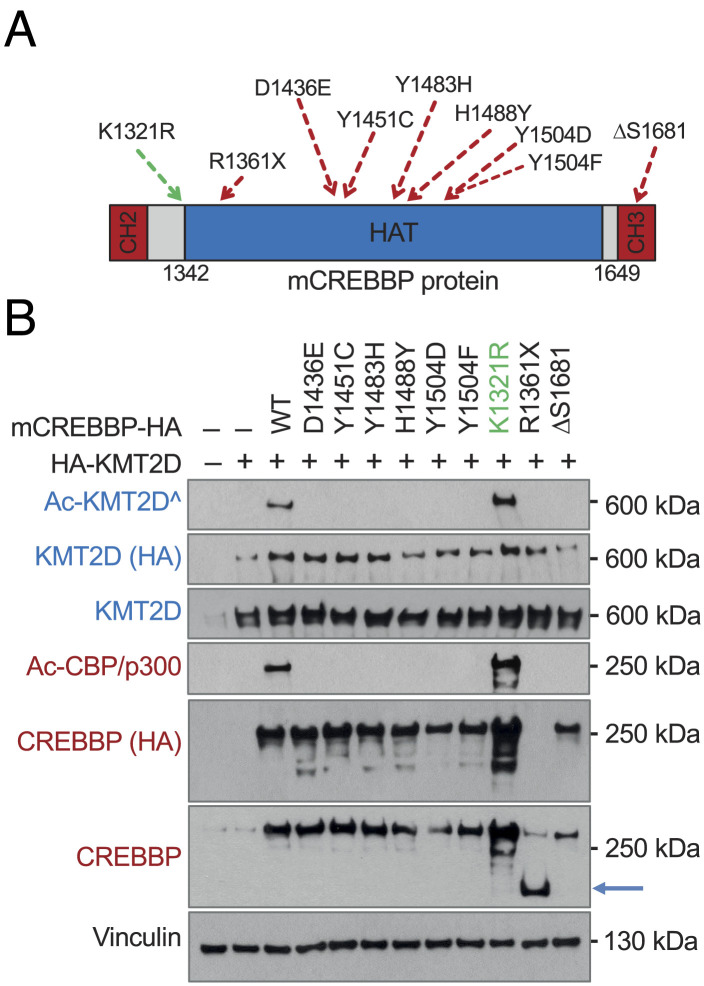
DLBCL-associated CREBBP HAT mutations abrogate the protein ability to acetylate KMT2D. (*A*) Schematic diagram of the CREBBP C-terminal HAT domain, with its flanking CH2 and CH3 (ZZ-TAZ2) domains. The position of common amino acid substitutions found in primary DLBCL samples and tested in the cotransfection assay is approximately indicated (numbering corresponds to mouse Crebbp). ΔS1681 is a recurrent in-frame deletion of a single serine. (*B*) Immunoblot analysis of CREBBP and KMT2D expression in HEK293T cells cotransfected with the indicated vectors. Acetylated KMT2D and CREBBP/p300 proteins are detected by a pan-AcK antibody (^), and vinculin controls for equal loading. Arrow indicates the exogenous truncated protein encoded by the R1361X construct (note that this protein is not detected by the HA antibody as the stop codon was introduced in the full-length sequence 5' to the C-terminal tag).

In order to test the consequences of the many CREBBP amino changes associated with FL and DLBCL on KMT2D acetylation, we used expression constructs for seven common missense HAT mutant alleles, as well as a recurrent in-frame deletion located C-terminal to the HAT domain (ΔS1681) and, as control, a HAT truncation mutant to perform transient cotransfection assays in 293T cells (Fig. 7*A*). With the expected exception of the K1321R change, which is located outside of the HAT domain, all mutant proteins investigated had lost their ability to acetylate KMT2D (Fig. 7*B*). Accordingly, KMT2D acetylation levels were significantly lower in native DLBCL cell lines carrying biallelic inactivation of CREBBP (SUDHL16) or lacking the expression of a full-length CREBBP protein (SUDHL10^CU^) compared to CREBBP wild-type cell lines (*SI Appendix*, Fig. S7). These observations are consistent with the results obtained in the engineered *CREBBP*^KO^ clones, providing support to the conclusion that KMT2D acetylation is impaired in CREBBP-mutant FL and DLBCL cases.

### Loss of KMT2D Acetylation Is Associated with Reduced H3K4 Monomethylation.

**Fig. 8.**
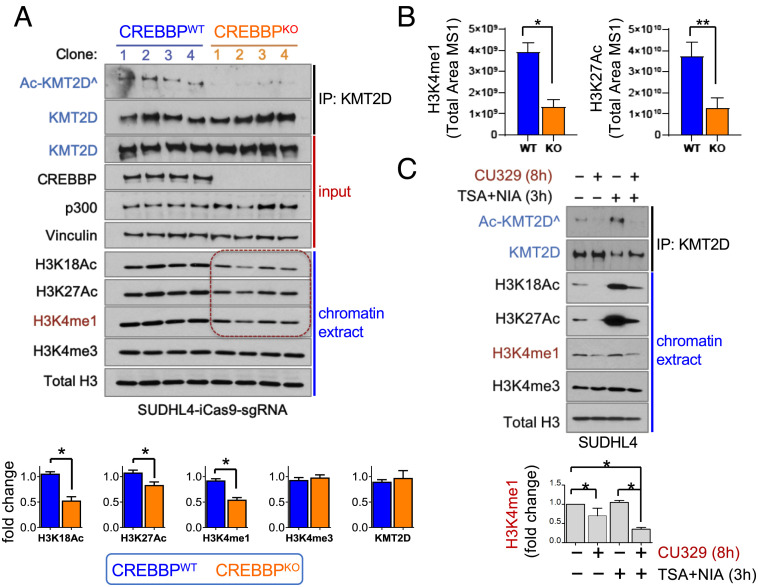
*CREBBP*-mutant DLBCL cells display significantly reduced H3K4me1 levels. (*A*) Immunoblot analysis of histone marks in chromatin extracts from isogenic CREBBP^WT^ and CREBBP^KO^ DLBCL clones treated with TSA/NIA (n = 4 each) (*Bottom*). Expression of CREBBP, KMT2D, and Ac-CREBBP/p300 in whole-cell extracts from the same cells is shown in the middle panels (input), while total and Ac-KMT2D in the IPs are in the top panels. Below the blots, quantification by densitometry using ImageJ (n = 4 clones/each; mean ± SD, with the WT levels arbitrarily set as 1; one representative experiment out of two that showed analogous results). (*B*) Relative abundance of H3K4me1 (TK(me1)QTAR) and H3K27Ac (KSAPATGGVKKPHR) in the same cells, assessed by mass spectrometry (n = 3 clones each; mean ± SD). (*C*) Immunoblot analysis of histone marks in SUDHL4 cells treated with TSA/NIA in the presence or absence of the specific CBP/p300 HAT inhibitor CU329 (*Bottom*). The levels of total and Ac-KMT2D in the KMT2D-IP are shown in the top panel (^, pan-AcK antibody). Quantification of H3K4me1 is displayed below the blots. In all panels, *P < 0.05 and ** *P *< 0.01, two-tailed Student’s *t* test. Only statistically significant *P* values are indicated.

To explore the functional impact of KMT2D acetylation in GC B cells, we first examined the pattern of H3K4 methylation in chromatin extracts obtained from isogenic SUDHL4 cell lines expressing low levels of acetylated KMT2D due to CREBBP disruption (n = 4 clones each, *CREBBP*^WT^ vs *CREBBP*^KO^) (Fig. 8*A*, *Top* and *Middle*). As expected, specific marks of CREBBP activity, like H3K27Ac and H3K18Ac, were significantly diminished in all *CREBBP*^KO^ clones (*P *< 0.05, Student’s *t* test) (Fig. 8*A*, chromatin extract; see *Bottom* panel for quantification). However, a measurable reduction was also seen in the levels of H3K4me1 by both western blot analysis and mass spectrometry (*P *< 0.05), whereas the amount of H3K4me3 and total histones was unchanged (Fig. 8 *A* and *B*). Analogously, global H3K4me1, but not H3K4me3, was significantly reduced in cells treated with the CU329 HAT inhibitor (Fig. 8*C*) (mean relative intensity: 1.0 vs 0.3, *P *< 0.05; *Bottom*). ChIP-seq analysis of H3K4me1 in the same cells (n = 2 clones each) confirmed significantly lower levels at ~9% of the peaks identified in control CREBBP^WT^ cells (N = 4072/47451; FDR < 0.05 and FC > 1.2) (*SI Appendix*, Fig. S8*A*). The affected regions were mostly represented by TSS-distal, intragenic and intergenic domains not decorated by H3K27Ac, which typically represent primed enhancers (*SI Appendix*, Fig. S8*B*). Collectively, these data are consistent with a role for CREBBP-mediated acetylation in sustaining the methyltransferase activity of KMT2D (*Discussion*). Of note, the reduction of H3K4me1 in CREBBP-deficient cells was milder than that observed upon complete ablation of KMT2D expression (*SI Appendix*, Fig. S8*C*), suggesting that CREBBP-mediated acetylation may act as a potentiating rather than as an essential mechanism for the fine-tuning of KMT2D activity and/or implying the existence of additional KMT2D structure–function roles.

## Discussion

The studies herein uncover a biochemical and functional interaction between CREBBP and KMT2D in GC-derived B cells and demonstrate a cooperative role for the disruption of these two genes in promoting the preneoplastic expansion of the GC B cell population (the normal counterpart of most human B cell lymphomas). GC hyperplasia is achieved by corrupting select enhancer networks that are distinct from those perturbed by each lesion alone and are linked to the response of GC B cells to environmental cues. These findings broaden the scope of CREBBP-mediated enhancer regulation beyond current knowledge based on its ability to acetylate H3K18 and H3K27, and have implications for the understanding and therapeutic targeting of the early phases of FL/DLBCL pathogenesis, where mutations inactivating these enzymes are coselected at high frequency.

Genetic lesions of *CREBBP* and *KMT2D* are considered founder events in the evolutionary history of FL/tFL and DLBCL, which are acquired by a long-lived common mutated ancestor cell prior to its final clonal expansion in the GC (10–13, 44). The synergistic effect of heterozygous *Crebbp/Kmt2d* loss in perturbing the GC response (a phenotype not observed when these genes are individually monoallelically deleted at this stage) and the identification of shared and specific CREBBP/KMT2D-bound chromatin domains offer a mechanistic explanation to the frequent concurrent selection of these alterations in lymphoma. In particular, our study indicates that codeletion of these two enzymes may be advantageous to tumor precursor cells by dysregulating a broad set of enhancers otherwise minimally sensitive to their individual inactivation. These enhancers are largely implicated in the delivery of signals that are required for cell fate decisions and immune surveillance checkpoints in the GC LZ, where B lymphocytes interact with the “immune niche” through multiple cell surface receptors (e.g. the BCR, TNFR, IFNR, and antigen presentation complexes). Consistently, the GC B cells of dHET mice showed a significant depletion in signatures associated with intermediate and LZ B cell subsets, particularly those reentering the DZ, and the desynchronization of genes linked to memory B cell commitment. The quantitative and qualitative changes identified here may thus create a permissive epigenetic environment for the persistence of GC B cells in this potentially deleterious environment, where they could evade T cell–dependent checkpoints and remain exposed to the mutagenic activity of AID in the context of a diminished DNA damage response (1).

Despite presenting a clear preneoplastic phenotype, *Kmt2d/Crebbp* dHET mice did not develop overt lymphomas even on 18 mo of follow-up (n = 0/17 vs 0/23 in littermate controls), indicating that perturbation of the epigenetic landscape alone is insufficient to drive full tumor formation and that additional genetic, epigenetic, and/or microenvironmental cues are necessary to lock these cells in a neoplastic state. This could be the case when GC-specific codeletion of *Crebbp/Kmt2d* is introduced in the FL-prone Bcl2-transgenic background, recapitulating the genetic makeup of FL/tFL and DLBCL. The fact that the gene expression signature disrupted in mouse dHET GC B cells can be back-tracked in human *CREBBP/KMT2D* comutated GCB-DLBCL supports the relevance of these findings to the human disease.

In addition to this cooperative functional interaction, the documentation of KMT2D as a direct substrate of CREBBP-mediated acetylation adds a new layer to the dynamic interplay between these epigenetic modifiers on chromatin. As major regulators of enhancer activity, KMT2D and CREBBP are thought to commission cell context–dependent enhancer networks through distinct epigenetic modifications of core histone tails, although more complex roles are emerging for these proteins beyond their catalytic activity (45). In particular, current models postulate that KMT2D is responsible for priming active E/SE regions prior to chromatin acetylation and enhancer activation by CREBBP (46, 47). Moreover, recent work has shown that KMT2D is required for full CREBBP activity at selected cell identity genes involved in the differentiation of adipocytes and myocytes (24). Conversely, a feedforward regulatory loop between KMT2D and p300 has been described in mouse ESCs as necessary to establish active enhancer landscapes (25). By demonstrating that CREBBP acetylates KMT2D and possibly modulates its activity, our findings support a mutual cooperativity between these two enzymes in the GC. Interestingly, CREBBP acetylation levels and total protein amounts were also increased in cells cotransfected with KMT2D (Fig. 5*D*), suggesting these two proteins may help each other through multiple mechanisms. Such biochemical interaction could serve as a rheostat that allows GC B cells to coordinately remodel their epigenome through graduated quantitative regulation, thus conferring epigenetic plasticity as they rapidly recirculate between the GC DZ and LZ before committing to post-GC differentiation (1, 48).

Our results show a correlation between CREBBP-mediated acetylation of KMT2D and histone H3K4me1, which was negatively affected in *CREBBP*^KO^ (KMT2D-Ac-defective) cells. Although these data are consistent with a role for acetylation in modulating the activity of KMT2D, additional studies (including mapping the critical KMT2D acetylation sites, among the many identified here, and the construction of acetylation-defective KMT2D polypeptides) will be needed to document whether reduced H3K4me1 in these cells reflects direct vs indirect effects, and to more broadly determine the biological consequences of impaired KMT2D acetylation. In particular, future efforts should aim to mechanistically dissect the impact of KMT2D acetylation on its catalytic vs noncatalytic function (45, 49), as well as on protein stability and the interaction with other proteins. We cannot exclude that lower levels of H3K4me1 in *CREBBP*^KO^ cells are secondary to the impairment of other (histone modifier) proteins involved in this reaction or to the reduced H3K27Ac imposed by CREBBP loss, which could render these histones a suboptimal substrate. However, the latter scenario is not supported by currently prevailing models that posit monomethylation is the first step in priming E/SEs for activation prior to acetylation, and that H3K27Ac is acquired almost exclusively in the context of preexisting H3K4me1 (50). Regardless of the underlying mechanism, one implication of our findings is that the loss of (CREBBP-mediated) KMT2D acetylation endows cells with a certain degree of KMT2D insufficiency in the absence of its direct genetic inactivation. This would be in line with recent observations suggesting that *CREBBP* mutations occur prior to *KMT2D* mutations, as reduced dosage of CREBBP may induce subtle changes before irreversible KMT2D mutational inactivation (13). Thus, one may envisage a stepwise model of lymphomagenesis whereby the common precursor cell progressively loses its ability to synchronize the activation of specific enhancer networks.

By demonstrating the physiological relevance of KMT2D acetylation in the context of the GC B cell compartment, our findings advance recent observations from a proteomic screen that nominated KMT2D among approximately 2,000 proteins composing the CREBBP/p300 acetylome of mouse embryonic fibroblasts (37). This effort was based on the dual knockout of *CREBBP* and *EP300* that, because of their extensive sequence and functional homology, are often treated as a single entity. Our data seem to suggest a preferential role for CREBBP over p300 in catalyzing this posttranslational modification (Fig. 6) and are in agreement with an increasing body of literature consolidating a functional separation between these KAT3 family members (42).

Finally, these findings have therapeutic implications for the management of both FL and DLBCL patients, as they provide additional rationale for the combinatorial targeting of CREBBP (e.g., by using HDAC3 small-molecule inhibitors) (42, 51) and KMT2D defects (e.g., by using KDM5 demethylase inhibitors) (52) in cases where both genes are mutated. Such an approach could be considered a specific strategy for eradicating the common precursor cell and thus a potential therapy for preventing relapse and transformation of FL into tFL/DLBCL.

## Methods

### Mouse Models and Strains.

The conditional *Crebbp* knockout and *Kmt2d* knockout mouse models were a generous gift of Drs. Paul Brindle (St Jude Children’s Hospital) and Kai Ge (NIDDK, NIH), respectively, and have been reported (16, 17, 53, 54). Mouse strains were backcrossed into *C57BL/6* background for over 6 generations and housed in a dedicated pathogen-free environment. Deletion of *Crebbp* and/or *Kmt2d* was directed to GC B cells by breeding mice with the *Cγ1-Cre* deleter strain (26) (in pure C57BL/6 background), followed by offspring intercrossing. All animal work was performed under protocols conformed to the National Cancer Institute guidelines and approved by the Columbia University Institutional Animal Care and Use Committee. Genotyping was performed by PCR analysis, and the protocols are available upon request.

### Mouse Immunizations.

For the analysis of T cell–dependent responses, age-matched 12- to 16-wk-old and 6-mo-old mice were immunized by intraperitoneal injection of the T cell–dependent antigen sheep red blood cells (SRBCs) (Cocalico Biologicals) (n = 500 million/mouse in PBS), followed by analysis after 10 d. Alternatively, mice were immunized by 200 μg 4-hydroxy-3-nitrophenylacetyl hapten conjugated to keyhole limpet hemocyanin (NP–KLH) (Biosearch Technologies) in Complete Freund’s Adjuvant (Sigma-Aldrich) and analyzed after 12 d. To achieve a higher yield of GC B cells for transcriptomic analyses, mice were immunized with two sequential SRBC injections (day 0, 1 × 10^8^ cells, and day 5, 1 × 10^9^ cells) and killed at day 12 for cell collection.

### Flow Cytometric Analysis of Mouse B Cell Subsets.

Multicolor flow cytometric analysis of the B lymphoid compartment was performed at 3 and 6 mo of age as previously reported (16) using 3 to 4 mice/genotype/experiment. Briefly, single cell suspensions prepared from mouse spleens and bone marrow were subjected to red blood cell lysis followed by staining for 20 min on ice, using different combinations of antigen-specific fluorescent-labeled antibodies (Dataset S5). For the detection of intracellular proteins, cells were fixed and permeabilized using BD Cytofix/Cytoperm™ (BD Biosciences, catalog #554714) following the manufacturer’s instructions and subsequently stained for 30 min at room temperature with the appropriate antibodies (Dataset S5b). Data were acquired on a FACSCanto™ II (BD Biosciences) and analyzed in FlowJo (TreeStar) using established gating strategies for the identification of different B cell subpopulations (16, 17, 55). To calculate the absolute numbers of cells within splenic B cell subsets, spleen fragments were weighed, and erythrocyte-depleted cell suspensions were counted by trypan blue exclusion using the Countess Automated Cell Counter (Thermo Fisher Scientific). The total number of counted splenic B cells was then multiplied by the fraction of each subpopulation, as identified by the cytofluorometric analyses, and is reported per mg of spleen.

### Sorting of Murine GC B Cells.

GC B cells were sorted from SRBC or NP-KLH immunized *Crebbp^HET^*, *Kmt2d^HET^*, *Crebbp/Kmt2d^dHET^,* and wild-type littermates (n = 3 mice each) as B220^+^CD95^+^PNA^hi^ live lymphocytes using a BD Influx^TM^ (Becton Dickinson). Cells were then washed in cold PBS 1X and used for downstream analyses.

### Chromatin Immunoprecipitation and Sequencing (ChIP-Seq).

ChIP-seq analysis of CREBBP and KMT2D was performed on 25 million cells/sample as previously described and has been reported in detail elsewhere (16, 17) (*SI Appendix*, *Supplementary Methods*). All experiments were performed in two biological replicates (two independent pools of sorted GC B cells), and raw data have been deposited to the GEO under accession nos. GSE67494 (56) and GSE89688 (57).

### Functional Annotation of CREBBP- or KMT2D-Bound Regions.

Regions bound by CREBBP or KMT2D were annotated as promoters if located within −2/+1 kb from the transcription start site (TSS) of an annotated gene, intragenic if distal to a TSS but within an annotated gene, and intergenic if distal to a TSS and located in nongenic regions, using the GRCh37 assembly. Active enhancers (Es) and superenhancers (SEs) were defined by ROSE as published (30) and confirmed based on the presence of overlapping H3K4me1 marks. In brief, ROSE identifies enhancers (Es) as all H3K27Ac peaks that do not overlap with known gene promoters (±2 kb from TSS, unless embedded in a larger H3K27Ac-positive chromatin domain), after concatenating those located within ±12.5 kb from each other, and then ranks them by their input-subtracted H3K27Ac signal. The cut point between enhancers and superenhancers was defined on the enrichment profile as the inflection point of H3K27Ac signal intensity vs the concatenated enhancer rank. CREBBP- or KMT2D-bound intragenic and intergenic regions were classified based on enhancer-associated chromatin marks as active (H3K4me1^+^/H3K4me3^−^/H3K27Ac^+^) or poised (H3K4me1^+^/H3K4me3^−^/H3K27Ac^-^) (58, 59). Read distribution plots around KMT2D-bound regions were created by centering a 3-kb window on the midpoint of KMT2D ChIP-seq peaks and dividing it into 100-bp bins. Histone mark reads found in the 6-kb window were used to produce a binned count distribution. Read counts were normalized to bins per million (BPM), and the average BPM was plotted. These data are reported in Fig. 2*C*. For the analysis of overlaps displayed in Fig. 2*E*, we used peaks mapping to GC-specific E/SEs (identified based on ROSE as described above) and decorated by H3K4me1 (KMT2D) or H3K27Ac (CREBBP). The significance of the overlap was determined by the hypergeometric distribution using all active GC E/SEs as denominator. Further details on gene assignment can be found in *SI Appendix*, *Supplementary Methods*.

### Transcription Factor Motif Discovery.

To identify TF binding motifs enriched in CREBBP/KMT2D-cobound chromatin domains, we used the HOMER motif discovery algorithm (homer2 version) using default parameters and both the de novo and known motif functions (60).

### RNA-Seq Analysis of Murine GC B Cells.

To identify differentially expressed genes in *Crebbp/Kmt2d* single and double heterozygous GC B cells, total RNA was extracted from sorted B220^+^CD95^+^PNA^hi^ splenocytes (n = 3 animals/genotype) using the NucleoSpin XS Kit (Macherey-Nagel, catalog #740902) as per manufacturer’s instructions and verified for integrity on a BioAnalyzer 2100 (Agilent). Samples (100 to 200 ng each) with RNA integrity numbers (RIN) >9 were processed to generate RNA-seq libraries using the Illumina TruSeq RNA Library Preparation Kit v2. High-throughput sequencing was performed on an Illumina NovaSeq 6000 instrument using a paired-end 150-bp protocol. RNA-seq reads were mapped to the Mus musculus (mm10/GRCm38) genome assembly using the hisat2 prebuilt genome index (61). Genome-mapped reads were aligned to exons on the mm10 transcriptome reference (62) based on the information in the genomic BAM files using featureCounts (v1.6.3) (63) to produce abundance tables. We sanitized the transcriptome references (exon only) by removing read-through genes, antisense elements, rRNA, and miRNA. We also filtered out chrY genes, imprinted chrX genes, pseudogenes and the immunoglobulin variable, diversity, and joining regions. Gene expression counts were subsequently normalized to produce transcript per million (TPM) mapped reads. Unsupervised clustering was performed on the top 500 genes with the highest SD across all samples using Pearson correlation with average linkage. Differentially expressed genes were determined using DESeq2 (64) separately for WT vs *Crebbp*^HET^, WT vs *Kmt2d*^HET^, and WT vs dHET, with the following filters: FDR < 0.05 (after Benjamini–Hochberg correction) and absolute fold change (FC) ≥ 1.2. Heat maps and expression plots were generated in R.

### Transient Transfections and Lentiviral Transductions.

HEK293T cells were transiently transfected using polyethylenimine (PEI) (Polysciences) as previously described (65). Briefly, DNA was diluted in 150 mM NaCl and incubated with PEI for 15 min at room temperature prior to addition to cells. Upon incubation at 37 °C for 7 to 8 h, the transfection medium was replaced with fresh complete medium, and cells were harvested 42 to 48 h after transfection. DNA amounts were adjusted to achieve comparable KMT2D protein levels in the absence or presence of CREBBP. For the transduction of DLBCL cells, viral supernatants were obtained in IMDM by transient transfection of HEK293T cells with lentiviral vectors along with a lentivirus packaging vector (∆8.9) and a plasmid-encoding vesicular stomatitis virus envelope glycoprotein (VSVg). Cells were then infected by spinoculation in two rounds and sorted after 48 h using the SH800 Cell Sorter (Sony Biotechnology) to obtain a pure population of infected GFP^+^ or RFP^+^ cells. Details on the expression constructs used are provided in *SI Appendix*, Fig. S5 *B*–*D*.

### Protein Extraction and Immunoprecipitation.

Whole-cell extracts were obtained from human naive and GC B cells, 293T cells, and DLBCL cell lines in the log phase of growth using NP-40 lysis buffer [50 mM Tris-HCl, pH 7.5, 150 mM NaCl, 0.2 mM EDTA, pH 7.0, 0.1% NP-40, 0.2% Triton X-100, 30 mM beta-glycerophosphate, 0.5 mM PMSF, 50 mM sodium fluoride, 1 mM sodium orthovanadate, and protease inhibitor cocktail (Sigma-Aldrich)] or Co-IP buffer (for immunoprecipitation: 50 mM Tris, pH 7.5, 250 mM NaCl, 1% Triton X-100, and 1 mM EDTA), according to previously described protocols (20). To detect the physical interaction of endogenous CREBBP and KMT2D, immunoprecipitation was performed from nuclear extracts prepared as reported in ref. 66. Histones were extracted using an acid extraction method; briefly, chromatin pellets were resuspended in 0.2 N HCl, incubated overnight at 4 °C, and cleared by centrifugation at 12,000 rpm for 10 min. For immunoprecipitation purposes, whole-cell or nuclear lysates were precleared with Protein G beads (GE Healthcare) for 1 h at 4 °C and incubated overnight at 4 °C with one of the following antibodies: anti-FLAG or anti-HA affinity beads (Sigma-Aldrich), anti-KMT2D antibody (Sigma-Aldrich, catalog #HPA035977), and anti-CREBBP antibody (Cell Signaling Technology, D6C5) (Dataset S5). Beads were washed five times in the same buffer, and immunocomplexes were eluted in the presence of 1 mg/mL FLAG or HA peptide (Sigma-Aldrich) or directly in SDS-PAGE sample-loading buffer.

### Western Blot Analysis.

Protein extracts or immunoprecipitates were resolved on NuPAGE Tris-Acetate 3 to 8% gels (for CREBBP, p300, and KMT2D) or Tris-Glycine 4 to 20% gels (for histone H3 and other low-MW proteins) (Life Technologies) at 4 °C and transferred to nitrocellulose membranes (GE Healthcare) according to the manufacturer’s instructions. After blocking for 1 h in PBS 1X with 0.05% Tween 20 and 5% dry fat milk, membranes were incubated with one of the following primary antibodies at 4 °C overnight: rabbit polyclonal anti-KMT2D (HPA035977, Sigma-Aldrich), rabbit monoclonal anti-CREBBP (D6C5), rabbit monoclonal anti-p300 (D2X6N), rabbit polyclonal antiacetylated lysine (9441), and rabbit polyclonal antiacetylated CBP (lysine 1535)/P300 (lysine 1499) (4771) (all from Cell Signaling Technology); mouse monoclonal anti-CRISPR/Cas9 antibody (7A9, EpiGentek, A-9000), mouse monoclonal anti-ß-actin (A5441, Sigma-Aldrich), mouse monoclonal anti-α-tubulin (clone B512, Sigma-Aldrich), rabbit polyclonal anti-H3K18Ac (Abcam, catalog #ab1191) and anti-H3K27Ac (Abcam, catalog #ab4729), and rabbit monoclonal anti-histone H3 (clone D1H2, Cell Signaling Technology). Detection was obtained by using the ECL Reagent or the SuperSignal West Dura Extended Duration Substrate (Thermo Fisher Scientific), followed by exposure to film (Kodak). Quantification of signal intensity was obtained in ImageJ (67), and values are expressed as fold differences relative to the reference (unedited or untreated) sample, set at 1, after normalization for the loading control.

### Identification of KMT2D Acetylation Sites by Mass Spectrometry.

To map the acetylated lysines in the KMT2D-fragment C region, proteins were affinity-purified from SUDHL4-F_2_H_2_-KMT2D-C and control SUDHL4-F_2_H_2_-EV cells by sequential immunoprecipitation using FLAG and HA beads, followed by HA peptide elution, separated on 4 to 12% gradient SDS-PAGE, and stained with SimplyBlue (Thermo Fisher Scientific). Protein gel slices were excised, and *in-gel* digestion was performed as previously described (68), with minor modifications (details on the approach and data analysis in *SI Appendix*, *Supplementary Methods*).

### Statistical Analysis.

To assess statistically significant differences between groups, *P* values were calculated with the Student’s *t* test (unpaired, two-tailed), Fisher’s exact test (unpaired, two-tailed), or one-way ANOVA with Bonferroni post hoc correction in GraphPad Prism v6.0. Statistical testing and samples sizes are indicated for each figure in the corresponding legend. Results were considered statistically significant at *P *< 0.05. Metrics and statistics used in the ChIP-seq, RNA-seq, and downstream pathway enrichment analyses are described in the corresponding Methods sections and figure legends. Statistics were calculated using R 4.2.2 and the SciPy Python library.

### Additional Methods.

Can be found in Supplementary Information.

## Supplementary Material

Appendix 01 (PDF)

Dataset S01 (XLSX)

Dataset S02 (XLSX)

Dataset S03 (XLSX)

Dataset S04 (XLSX)

Dataset S05 (XLSX)

Dataset S06 (PDF)

## Data Availability

The raw CREBBP and KMT2D ChIP-seq data from human GC B cells are available in the GEO database as series GSE89688 (57) and GSE67494 (56). The mouse RNA-seq data have been deposited in the GEO database under accession no. GSE220255 (69). The human RNA-seq and Affymetrix microarray data for DLBCL were obtained from dbGaP (accession no. phs000532.v11.p2) (70) and GEO (accession no. GSE98588) (71). All study data are included in the article and/or *SI Appendix*.
