# Supplementary material for: KMT2D acetylation by CREBBP reveals a cooperative functional interaction at enhancers in normal and malignant germinal center B cells
Source: Proc Natl Acad Sci U S A. 2023 Mar 9;120(11):e2218330120. doi: 10.1073/pnas.2218330120 (PMC10089214; doi:10.1073/pnas.2218330120)
Supplement: Supplementary file 1 — Appendix 01 (PDF) [file pnas.2218330120.sapp.pdf]

SUPPLEMENTARY INFORMATION

KMT2D acetylation by CREBBP reveals a cooperative functional interaction in normal and malignant germinal center B cells

Sofija Vlasevska[1], Laura Garcia-Ibanez[1], Romain Duval[1^], Antony B. Holmes[1], Rahat Jahan[1], Bowen Cai[1], Andrew Kim[1], Tongwei Mo[1], Katia Basso[1,2], Rajesh K. Soni[3,4], Govind Bhagat[2], Riccardo Dalla-Favera[1,2,4-6] and Laura Pasqualucci[1,2,4*]

1 Institute for Cancer Genetics, Columbia University, New York, NY 10032, USA
2 Department of Pathology and Cell Biology, Columbia University, New York, NY, 10032, USA
3 Proteomics and Macromolecular Crystallography Shared Resource, Columbia University, New York, NY, 10032, USA
4 Herbert Irving Comprehensive Cancer Center, Columbia University, New York, NY, 10032, USA
5 Department of Genetics & Development, Columbia University, New York, NY, 10032, USA
6 Department of Microbiology & Immunology, Columbia University, New York, NY, 10032, USA
^ Present address: Département Centre National de Référence pour les Groupes Sanguins, Etablissement Français du Sang-Ile-de-France, 75012 Paris, France

* Correspondence: lp171@cumc.columbia.edu

CONTENT

SUPPLEMENTARY METHODS

SUPPLEMENTARY FIGURES

Supplementary Figure 1. *Crebbp/Kmt2d* dHET mice show enlarged GCs with altered polarization and SHM load

Supplementary Figure 2. CREBBP/KMT2D co-occupied regions are enriched in ETS TF binding motifs.

Supplementary Figure 3. Differentially expressed programs in *Crebbp/Kmt2d* dHET GC B cells and CREBBP/KMT2D co-mutated human DLBCL

Supplementary Figure 4. Experimental approach for the analysis of KMT2D acetylation in human GC-derived B cells

Supplementary Figure 5. The KMT2D acetylated region maps to amino acids 2289-3170

Supplementary Figure 6. Semi-purified proteins used in the *in vitro* acetylation assays

Supplementary Figure 7. KMT2D acetylation levels are reduced in CREBBP-mutated DLBCL cell lines.

Supplementary Figure 8. ChIP-seq analysis of H3K4me1 in CREBBP-deficient DLBCL cells.

SUPPLEMENTARY METHODS

Cell lines. The human DLBCL cell lines SUDHL4, OCI-Ly7, SUDHL16, and SUDHL10[CU1], and their isogenic *CREBBP*^{KO} and *EP300*^{KO} derivatives were grown in Iscove's modified Dulbecco's medium (IMDM) supplemented with 10% fetal calf serum (FCS), 100 U/ml penicillin and 100 µg/ml streptomycin (see Ref[2] for details on the construction and validation of the isogenic CREBBP^{KO} and EP300^{KO} SUDHL4 and Ly7 cell lines). HEK293T cells (American Type Culture Collection) were grown in Dulbecco's modified Eagle medium (DMEM) supplemented with 10% FCS, 100 U/ml penicillin and 100 µg/ml streptomycin. Cells were maintained at 37°C in humidified incubators under 5% CO_2. All cell lines tested negative for Mycoplasma contamination and were verified for identity by STR profiling and/or by analysis of somatic single nucleotide variants, as obtained by whole genome sequencing. Where indicated, TSA (1µM)(Sigma-Aldrich, cat#T8552) and NIA (5mM) (Sigma-Aldrich, cat#N3379) were added to the culture for 3 h prior to harvesting.

Purified human GC and naïve B cells. Human GC centroblasts and naïve B cells were isolated from reactive tonsils as described[3]. Tonsils were obtained as residual material from routine tonsillectomies at the Children's Hospital of Columbia-Presbyterian Medical Center, in compliance with Regulatory Guideline 45 CFR 46.101 (b)(4) for Exempt Human Research Subjects of the US Department of Health and Human Services, and according to protocols approved by the Institutional Ethics Committee.

Expression constructs and sgRNA design. The full-length HA-tagged KMT2D and mCREBBP expression constructs (wild type and point-mutant derivatives) have been reported previously[4,5]. Plasmids expressing the full length human CREBBP protein (wild type and R1446L mutant) were generated by subcloning the CREBBP coding sequence into the pCMV-Tag2A vector, in frame with the FLAG tag (Agilent Technologies). Plasmids expressing a double tagged (Flag-Flag-HA-HA, abbreviated as F_2H_2) full-length KMT2D protein and 5 deletion mutants (A-E) tiled across the 5537AA long KMT2D, such that each fragment overlaps its flanking fragments by ~ 300bp, were obtained as a kind gift by Dr. Luca Busino, Perelman School of Medicine at the University of Pennsylvania (Supplementary Figure 5A). The pCDNA5-F_2H_2-KMT2D-ΔC construct lacking AA 2289-3170 was derived from the full-length pCDNA5-F_2H_2-KMT2D through sequential subcloning and site directed mutagenesis. For the construction of isogenic cell lines, the inducible Cas9 expression construct pCW-Cas9 (Addgene: #50661), pLKO5-sgRNA-EFS-GFP (Addgene: #57822), pLKO5-sgRNA-EFS-tRFP (Addgene: #57823), and pHIV-Zsgreen (Addgene: #18121) were acquired from Addgene [6,7]. Benchling (http://benchling.com) was used to design sgRNAs targeting active domains of the human *CREBBP*, *EP300* or *KMT2D* genes, as well as a neutral control region in the *PPP1R12C* intron 1 (at least two independent sgRNAs/gene). SgRNAs were then cloned into pLKO5 vectors as described[8]. The pHIV-Zsgreen-F_2H_2-KMT2D fragment C was obtained by subcloning the blunt ended Xba-NheI fragment of pCDNA5-F_2H_2-KMT2D-C.

Generation of isogenic DLBCL cell lines. The isogenic *CREBBP/EP300*^{WT}, *CREBBP*^{KO} and *EP300*^{KO} DLBCL cell lines SUDHL4 and Ly7 are described in detail in previous

work[2]. Briefly, cells were engineered to express an inducible Cas9 (iCas9) protein by lentiviral transduction using a standard protocol[5], followed by selection in complete IMDM medium containing puromycin (1mg/ml) to obtain single cell-derived clones, which were screened for high levels of Cas9 expression. Two independent clones displaying similar Cas9 induction efficiency were selected for delivery of lentiviral vectors carrying the sgRNA of interest, tagged with GFP or RFP (n=2 independent sgRNAs per gene, and 2 control sgRNAs directed against a neutral region of the genome). GFP and/or RFP-positive populations were isolated in a SH800 cell sorter (Sony Biotechnology) and single cell plated 3 days after doxycycline induction of Cas9 expression, in order to isolate *EP300*-null or *CREBBP*-null clones. Disruption of the target gene was verified by PCR amplification and direct sequencing, followed by inspection of the chromatograms both manually and using the Crisp-ID tool (http://crispid.gbiomed.kuleuven.be/)[9]. Clones carrying bi-allelic frameshift mutations were then expanded and confirmed to lack expression of the target gene by immunoblotting, with the sgNeutral edited cells as control. To map the KMT2D acetylation sites, SUDHL4 cells were engineered to stably express the F_2H_2-KMT2D-C polypeptide (or empty vector as control) by lentiviral transduction, followed by sorting of the GFP^+ population.

Small molecule CREBBP/p300 inhibitor. The specific CREBBP/p300 HAT inhibitor CU329 was synthesized as reported[2] and tested for on-target activity by immunoblotting of CREBBP/p300 self-acetylation, H3K18Ac and H3K27Ac[2].

Histological and immunohistochemical analysis of mouse lymphoid tissues. Histological analysis of mouse lymphoid organs was performed on 3μm-thick FFPE tissue sections, stained with Hematoxylin & Eosin (Thermo Scientific) according to standard procedures. The following primary antibodies were used for immunohistochemical analysis: anti-Bcl6 (1:300) (N3, rabbit polyclonal, Santa Cruz Biotechnology); biotin-conjugated anti-PNA (1:200) (Vector Laboratories); biotin-conjugated anti-B220 (1:400) (RA3-6B2, rat monoclonal, Pharmingen 553086) and anti-CD3 (1:800) (SP7, rabbit monoclonal, NeoMarkers RM9107)(Dataset S05b). A polymer-enhanced HRP-conjugated secondary antibody (for CD3 and BCL6; EnVision system, Agilent-Dako) or alkaline phosphatase (AP)-conjugated streptavidin (for B220 detection; Vector Laboratories) were then used prior to detection with either AEC substrate (HRP-conjugates, red color)(Sigma-Aldrich) or NBT/BCIP substrate (AP-conjugates, blue color)(Roche). GC number, size and overall area were calculated using the ImageJ software on scanned images (3 sections/mouse) obtained with a Leica SCN400 slide scanner[10].

Analysis of tumor formation. Animals in the tumor cohort were subjected to chronic immunization by i.p. injection of SRBC every two months, until 12 months of age, monitored for tumor incidence and survival twice/week over a period of 18 months, and sacrificed for analysis when visibly ill or at the study endpoint, according to protocols approved by the Columbia University Institutional Animal Care and Use Committee. Both females and males were included in the experiments, and a sample size of ≥23 animals/genotype was calculated to ensure over 80% power to detect differences in tumor formation at a $P<0.05$ significance. No randomization was used.

Chromatin Immunoprecipitation and sequencing (ChIP-Seq). Chromatin Immunoprecipitation (ChIP) was performed on 25 million cells/sample as previously described[5]. Briefly, cells were cross-linked with 1% formaldehyde for 10 min at RT, quenched by the addition of glycine to a final concentration of 0.125 M, and frozen. The TruChIP High Cell Chromatin Shearing kit with SDS (Covaris) was used for cell lysis and nuclei isolation, followed by sonication in an S220 ultrasonicator (Covaris, Woburn, MA) to a chromatin fragment size distribution of 200-500 bp. Sheared chromatin was incubated overnight with 4μg of the following antibodies, as reported: anti-H3K27Ac (Active Motif, cat#39133); anti-KMT2D (Sigma-Aldrich, cat#HPA035977); anti-CREBBP (Santa Cruz, cat#sc-369); anti-H3K4me1 (Abcam, cat#ab8895), and anti-H3K4me3 (Abcam, cat#ab8580). The immune-complexes were collected with protein A magnetic beads over a 4h incubation, and washed sequentially at increasing stringency before reverse cross-linking. Following RNAse and proteinase K treatment, DNA fragments were purified using the MiniElute Reaction Clean Up Kit (Qiagen) and quantified by Quant-iT PicoGreen dsDNA Reagent (Life Technologies). The specificity of the antibodies against H3K27Ac, H3K4me1, and H3K4me3 has been extensively documented (ENCODE Project: http://genome.ucsc.edu/ENCODE/antibodies.html; and http://www.activemotif.com). Barcoded ChIP-seq libraries were constructed starting from 4 ng of immunoprecipitated or input DNA as reported[4,11], quantified using the KAPA SYBR FAST Universal qPCR Kit (KAPA Biosystems), normalized to 15nM, and pooled for sequencing on an Illumina HiSeq 4000 instrument as paired-end 150 bp reads, obtaining on average 25×10^6 reads/sample.

ChIP-seq analysis. Sequencing data were processed according to the default Illumina pipeline using Casava V1.8, and raw reads were mapped to the human genome GRC37 assembly using the Bowtie2 aligner v2.1.0[12], allowing up to two mismatches. Duplicate reads (i.e., reads of identical length mapping to exactly the same genomic locations) were removed with SAM tools v0.1.19 using the rmdup option[13], and the remaining reads were normalized to total reads aligned; data are displayed as read counts per million mapped reads. Peaks were identified using ChIPseeqer v2.0[14], enforcing a minimum fold change of 2 between ChIP and input reads, a minimum peak width of 100 bp, and a minimum distance of 100 bp between peaks. The *P* value threshold for statistical significance of peaks was set at 10^{-15} for H3K4me1, H3K4me3, and H3K27Ac, 10^{-12} for CREBBP, and 10^{-5} for KMT2D. Peaks overlapping with the Encode Blacklist or an internal manually curated signal artifact blacklist were discarded. Only peaks (regions) detected in both biological replicates (i.e. overlapping peaks) were considered for downstream analyses, except for KMT2D where, because of the lower ChIP efficiency, we used the union list of peaks identified in the two samples (CB4 and CB6). H3K4me1 peaks located within ±2 kb from each other and H3K27Ac peaks located within ±12.5 kb were stitched using the ROSE algorithm[15], unless mapping around a transcription start site (TSS) (-2/+1kb) and not embedded in a SE. Peaks were then annotated using a custom script.

Assignment of KMT2D- and CREBBP-bound regions to genes. To assign intergenic peaks to candidate target genes, we first defined topologically associating domains (TADs) based on Hi-C data of human GC B cells (SRA:SRP077918)[4]. KMT2D and CREBBP

peaks mapping to E/SEs were then assigned to the nearest (distance from peak center to TSS) transcriptionally active (expression in GC B cells displaying histone activation marks in the linked E/SE, by RNA-seq and ChIP-Seq respectively) gene located within the same TAD as the most likely candidate target.

Pathway enrichment analysis and GSEA. To determine whether genes bound by CREBBP and/or KMT2D in human GC B cells were enriched in annotated functional categories, we used a hypergeometric test with a Benjamini-Hochberg false discovery rate correction, assessing the significance of the overlap between the list of genes occupied at E/SEs (top 500, based on ChIP-Seeker *P* value) and pre-defined gene collection lists provided in the MSigDB (https://www.gsea-msigdb.org/gsea/msigdb/annotate.jsp) (C2, C6, C7, H) or in the SignatureDB (https://lymphochip.nih.gov/signaturedb/). Pathways with a significant *P* value (FDR <0.05 after Benjamini-Hochberg correction) and relevance to B cell biology were retained and are listed in Dataset S02.

For the GSEA of transcriptomic profiles obtained from murine GC B cells, we used previously defined DZ and LZ signatures (Victora et al., 2012) as well as a curated list of gene sets from the MSigDB C2-CP collection (KEGG, BIOCARTA and REACTOME), the Signature DB, and the 13 GC clusters identified in our single cell transcriptomic analysis of human GC B cells[16], as described.

To determine enrichment of the murine signature (i.e. the set of genes significantly downregulated in dHET vs WT GC B cells) in human DLBCL cases, GSEA was performed using transcriptomic profiles from two independent, genetically characterized DLBCL datasets: the NCI-DLBCL cohort (n = 481 samples with RNA-seq data, labeled as

“DLBCL”, including 138 GCB-, 243 ABC- and 100 unclassified-DLBCLs, available as dbGaP Accession No. phs000532.v11.p2)[17] and the DFCI cohort (n = 136 samples analyzed by Affymetrix microarrays, including 54 GCB-, 62 ABC- and 20 unclassified-DLBCLs available as GEO Accession No. GSE98588)[18].

In-gel **digestion and mass spectrometric characterization of KMT2D acetylation.** Gel slices were washed with 1:1 acetonitrile and 100mM ammonium bicarbonate for 30 min, then dehydrated with 100% acetonitrile for 10 min until shrunk. Upon removal of excess acetonitrile, slices were dried in a speed vacuum for 10 minutes at room temperature, reduced with 5 mM DTT for 30 min at 56 ^{o}C in an air thermostat, cooled down to room temperature, and alkylated with 11 mM IAA for 30 min with no light. After washing with 100 mM of ammonium bicarbonate and 100% acetonitrile for 10 min each, excess acetonitrile was removed and the gel slices were dried in a speed-vacuum, re-hydrated in a solution of 25 ng/µl trypsin in 50 mM ammonium bicarbonate for 30 min on ice, and digested overnight at 37 ^{o}C in an air thermostat. Digested peptides were collected and further extracted in extraction buffer (1:2 ratio of 5% formic acid: acetonitrile) at high speed in an air thermostat. The supernatants from both extractions were combined and dried in a speed vacuum. Peptides were dissolved in 3% acetonitrile/0.1% formic acid.

Liquid chromatography with tandem mass spectrometry (LC-MS/MS). Desalted peptides were injected in an EASY-Spray™ PepMap™ RSLC C18 50cm X 75µm ID column (Thermo Scientific) connected to an Orbitrap Fusion™ Tribrid™ (Thermo Scientific). Peptides elution and separation were achieved at a non-linear flow rate of 250

nl/min using a gradient of 5 to 30% of buffer B (0.1% (v/v) formic acid, 100% acetonitrile) for 110 minutes, maintaining the temperature of the column at 50 °C during the entire experiment. Survey scans of peptide precursors are performed from 350 to 1500 *m/z* at 120K full width at half maximum (FWHM) resolution (at 200 *m/z*), with a 1 x 10^6 ion count target and a maximum injection time of 60 ms. After a survey scan, MS/MS was performed on the most abundant precursors, i.e., those exhibiting a charge state from 2 to 6 of greater than 5 x 10^3 intensity, by isolating them in the quadrupole at 1.6 Th. The higher energy collisional dissociation (HCD) with set at 30% collision energy and detected the resulting fragments scan in the Orbitrap. The automatic gain control (AGC) target for MS/MS was set to 5 x 10^4 and the maximum injection time was limited to 54ms. The dynamic exclusion was set to 30s with a 10-ppm mass tolerance around the precursor and its isotopes, and monoisotopic precursor selection was enabled.

LC-MS/MS data analysis. Raw mass spectrometric data were analyzed using the Proteome Discoverer (PD) 2.4 software at default settings with a few modifications. PD was set up to search the reference human proteome database with trypsin digestion allowing up to 2 missed cleavages. Carbamidomethylation of cysteine (C) was set as a fixed modification, and oxidation of methionine (M), acetylation of the protein N-terminus, K acetylation, and deamination for asparagine or glutamine (NQ) were set as variable modifications. The precursor ion mass tolerance was set to 10 ppm, and the product ion mass tolerance was set to 0.6 Da. A decoy database search was performed to determine the peptide false discovery rate (FDR) with the Target Decoy PSM Validator module. A 1% peptide FDR threshold was applied. Peptides groups table were used to identify the

acetylated (K) sites abundance.

H3K4me1 quantification by Tandem Mass Spectrometry. Histone post-translational modifications in isogenic SUDHL4-CREBBP-WT and SUDHL4-CREBBP-KO cell lines (n=4 clones each) were characterized by chemical derivatization and tandem mass spectrometry, as follows. Total histones were isolated from cells in exponential growth with the EpiQuik Total Histone Extraction kit (Epigentek) according to the manufacturer's instructions, visualized by Coomassie staining, recovered by precipitation with inclusion of salt[19], and subjected to chemical derivatization and trypsin digestion as described[20]. Briefly, histones (10µg) reconstituted in 20µl deionized water were mixed with 20 µL of 50 mM NH_4HCO_3 buffer (pH 8.0). Histone propionylation was performed by adding 10 µl acetonitrile: propionic anhydride (3:1) solution to each sample and mixing with 8 µL NH_4OH to re-establish the pH 8.0. Samples were incubated at room temperature for 15 minutes, dried via vacuum centrifugation, and subjected to a second round of propionylation. Trypsin digestion (20:1 protein:enzyme ratio) was performed overnight at 37 ^{0}C in 50 mM NH_4HCO_3, followed by propionylation of the N-termini of the resulting peptides. Samples were vacuum dried and desalted prior to LC-MS/MS analysis by using in-house packed SDB-RPS StageTips followed by vacuum centrifugation.

Dried peptides were dissolved in 10 µl of 3% acetonitrile/ 0.1% formic acid, and 500 ng were analyzed on an Orbitrap Fusion mass spectrometer coupled to a Dionex Ultimate 3000 (ThermoFisher Scientific) using the PRM method[21]. Peptides were separated on an EASY-Spray C18 25cm column (Thermo Scientific). Peptide elution and separation were achieved at a non-linear flow rate of 300 nl/min using a gradient of 5%-

30% of buffer B (0.1% (v/v) formic acid, 100% acetonitrile) for 100 minutes, maintaining the column temperature at 40 °C during the entire experiment. The Thermo Scientific Orbitrap Fusion Lumos Tribrid Mass Spectrometer was used for peptide tandem mass spectroscopy (MS/MS). MS data were acquired with two combined scan events corresponding to a full scan and a tMS^2 method targeting the specific H3 histone-modified peptides. The target value for the full scan MS spectra was 4×10^5 ions in the 350–650 *m/z* range with a maximum injection time of 50 ms and resolution of 120,000 at 200 *m/z*. The tMS^2 method were acquired with an isolation width of 1.6 *m/z*, a resolution of 60,000 on an Orbitrap, a target AGC value of 5.0×10^4, and fragmented by higher-energy C-trap dissociation with a normalized collision energy of 27 eV and a maximum injection time of 118 ms.

For data processing, raw MS files were imported and analyzed in the Skyline software v22.2.0.312 to generate XIC and perform peak integration with Savitzky-Golay smoothing[22]. The area under the curve of selected precursor ions was summed to determine the abundance of the respective H3 histone modified peptides. All Skyline peak assignments were manually confirmed.

REFERENCES TO SUPPLEMENTARY METHODS

1 Epstein, A. L. *et al.* Biology of the human malignant lymphomas. IV. Functional characterization of ten diffuse histiocytic lymphoma cell lines. *Cancer* **42**, 2379-2391, doi:10.1002/1097-0142(197811)42:5<2379::aid-cncr2820420539>3.0.co;2-4 (1978).

2 Meyer, S. N. *et al.* Unique and Shared Epigenetic Programs of the CREBBP and EP300 Acetyltransferases in Germinal Center B Cells Reveal Targetable Dependencies in Lymphoma. *Immunity* **51**, 535-547 e539, doi:10.1016/j.immuni.2019.08.006 (2019).

3 Klein, U. *et al.* Transcriptional analysis of the B cell germinal center reaction. *Proceedings of the National Academy of Sciences of the United States of America* **100**, 2639-2644 (2003).

4 Zhang, J. *et al.* Disruption of KMT2D perturbs germinal center B cell development and promotes lymphomagenesis. *Nat Med* **21**, 1190-1198, doi:10.1038/nm.3940 (2015).

5 Zhang, J. *et al.* The CREBBP Acetyltransferase Is a Haploinsufficient Tumor Suppressor in B-cell Lymphoma. *Cancer Discov* **7**, 322-337, doi:10.1158/2159-8290.CD-16-1417 (2017).

6 Heckl, D. *et al.* Generation of mouse models of myeloid malignancy with combinatorial genetic lesions using CRISPR-Cas9 genome editing. *Nature biotechnology* **32**, 941-946, doi:10.1038/nbt.2951 (2014).

7 Wang, T., Wei, J. J., Sabatini, D. M. & Lander, E. S. Genetic screens in human cells using the CRISPR-Cas9 system. *Science* **343**, 80-84, doi:10.1126/science.1246981 (2014).

8 Sanjana, N. E., Shalem, O. & Zhang, F. Improved vectors and genome-wide libraries for CRISPR screening. *Nature methods* **11**, 783-784, doi:10.1038/nmeth.3047 (2014).

9 Dehairs, J., Talebi, A., Cherifi, Y. & Swinnen, J. V. CRISP-ID: decoding CRISPR mediated indels by Sanger sequencing. *Scientific reports* **6**, 28973, doi:10.1038/srep28973 (2016).

10 Schindelin, J., Rueden, C. T., Hiner, M. C. & Eliceiri, K. W. The ImageJ ecosystem: An open platform for biomedical image analysis. *Molecular reproduction and development* **82**, 518-529, doi:10.1002/mrd.22489 (2015).

11 Reddy, A. *et al.* Genetic and Functional Drivers of Diffuse Large B Cell Lymphoma. *Cell* **171**, 481-494 e415, doi:10.1016/j.cell.2017.09.027 (2017).

12 Langmead, B. & Salzberg, S. L. Fast gapped-read alignment with Bowtie 2. *Nature methods* **9**, 357-359, doi:10.1038/nmeth.1923 (2012).

13 Li, H. *et al.* The Sequence Alignment/Map format and SAMtools. *Bioinformatics* **25**, 2078-2079, doi:10.1093/bioinformatics/btp352 (2009).

14 Giannopoulou, E. G. & Elemento, O. An integrated ChIP-seq analysis platform with customizable workflows. *BMC bioinformatics* **12**, 277, doi:10.1186/1471-2105-12-277 (2011).

15 Whyte, W. A. *et al.* Master transcription factors and mediator establish super-enhancers at key cell identity genes. *Cell* **153**, 307-319, doi:10.1016/j.cell.2013.03.035 (2013).

16 Holmes, A. B. *et al.* Single-cell analysis of germinal-center B cells informs on lymphoma cell of origin and outcome. *J Exp Med* **217**, doi:10.1084/jem.20200483 (2020).
17 Schmitz, R. *et al.* Genetics and Pathogenesis of Diffuse Large B-Cell Lymphoma. *N Engl J Med* **378**, 1396-1407, doi:10.1056/NEJMoa1801445 (2018).
18 Chapuy, B. *et al.* Molecular subtypes of diffuse large B cell lymphoma are associated with distinct pathogenic mechanisms and outcomes. *Nat Med* **24**, 679-690, doi:10.1038/s41591-018-0016-8 (2018).
19 Nickerson, J. L. & Doucette, A. A. Rapid and Quantitative Protein Precipitation for Proteome Analysis by Mass Spectrometry. *J Proteome Res* **19**, 2035-2042, doi:10.1021/acs.jproteome.9b00867 (2020).
20 Sidoli, S., Bhanu, N. V., Karch, K. R., Wang, X. & Garcia, B. A. Complete Workflow for Analysis of Histone Post-translational Modifications Using Bottom-up Mass Spectrometry: From Histone Extraction to Data Analysis. *J Vis Exp*, doi:10.3791/54112 (2016).
21 Gallien, S., Kim, S. Y. & Domon, B. Large-Scale Targeted Proteomics Using Internal Standard Triggered-Parallel Reaction Monitoring (IS-PRM). *Mol Cell Proteomics* **14**, 1630-1644, doi:10.1074/mcp.O114.043968 (2015).
22 MacLean, B. *et al.* Skyline: an open source document editor for creating and analyzing targeted proteomics experiments. *Bioinformatics* **26**, 966-968, doi:10.1093/bioinformatics/btq054 (2010).

Figure S1

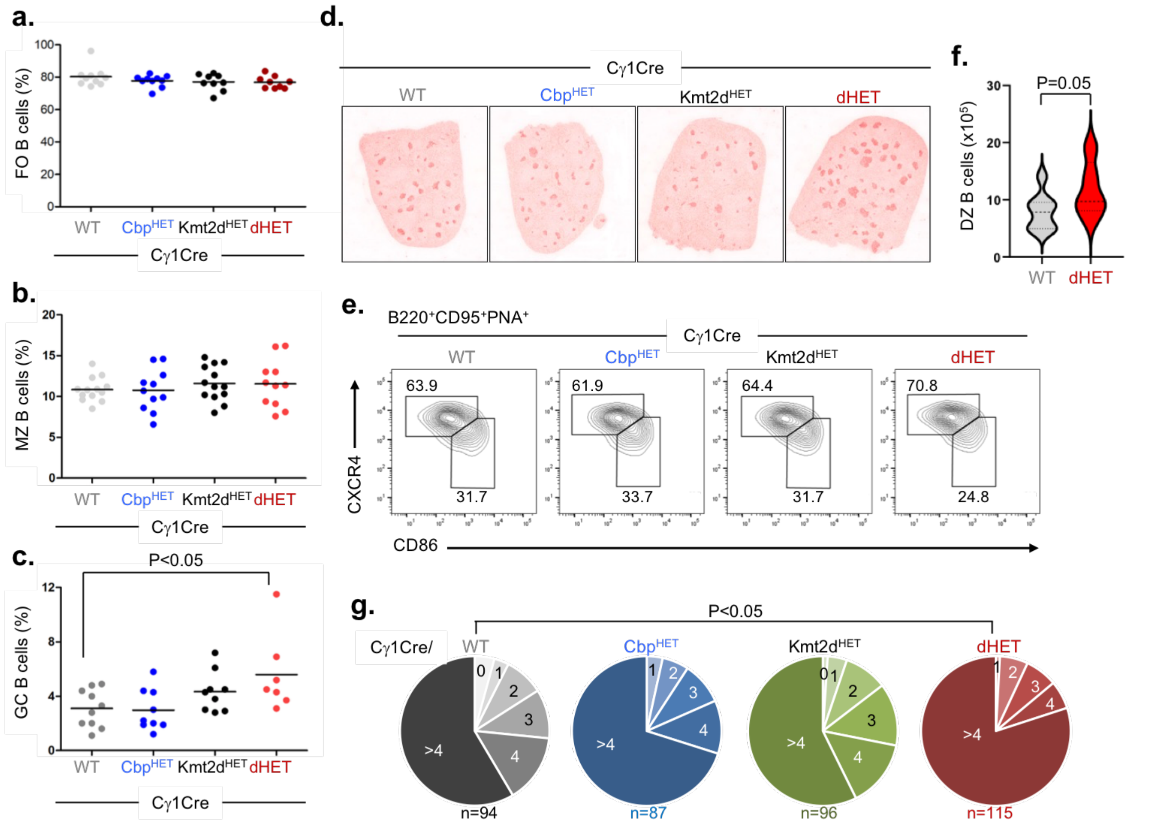

Supplementary Figure 1. *Crebbp/Kmt2d* dHET mice show enlarged GCs with altered polarization and SHM load. a, Percentage of follicular (FO) B cells (B220$^+$IgM$^+$IgD$^+$) in mice of the indicated genotypes. **b,** Percentage of marginal zone (MZ) B cells (B220$^+$IgM^{hi}IgD^{low}) in mice of the indicated genotypes. **c,** Percentage of GC B cells in SRBC_immunized mice of the indicated genotypes (data shown are from 3 representative experiments with comparable immunization efficiencies). Statistically significant differences are calculated by one-way ANOVA with Bonferroni correction and only significant values are shown. **d.** BCL6 IHC staining of spleen sections from representative mice of the indicated genotypes, analyzed 10 days after SRBC immunization. **e.** Representative FACS contour plots from GC B cells stained with the DZ and LZ markers CXCR4 and CD86 (data are gated on B220$^+$CD95$^+$PNA$^+$ cells). **f.** Absolute GC DZ B cell numbers in mice of the indicated genotypes (per mg of spleen) (n= 8 WT and 5 dHET from 2 representative experiments with comparable immunization efficiencies; differences in LZ B cells did not reach significance). **g.** Distribution of GC-B cell derived, V186.2-rearranged *IG* sequences showing different mutation loads. Numbers inside the pie refer to the total number of mutations in the V region, and the size of the segment is proportional to the fraction of unique rearrangements displaying the indicated mutational load. The total number of unique rearrangements analyzed (i.e. unique CDR3 sequences) is provided below for each of the four genotypes, and only statistically significant *P* values (vs WT) are indicated (two-tails Student's t-test).

Figure S2

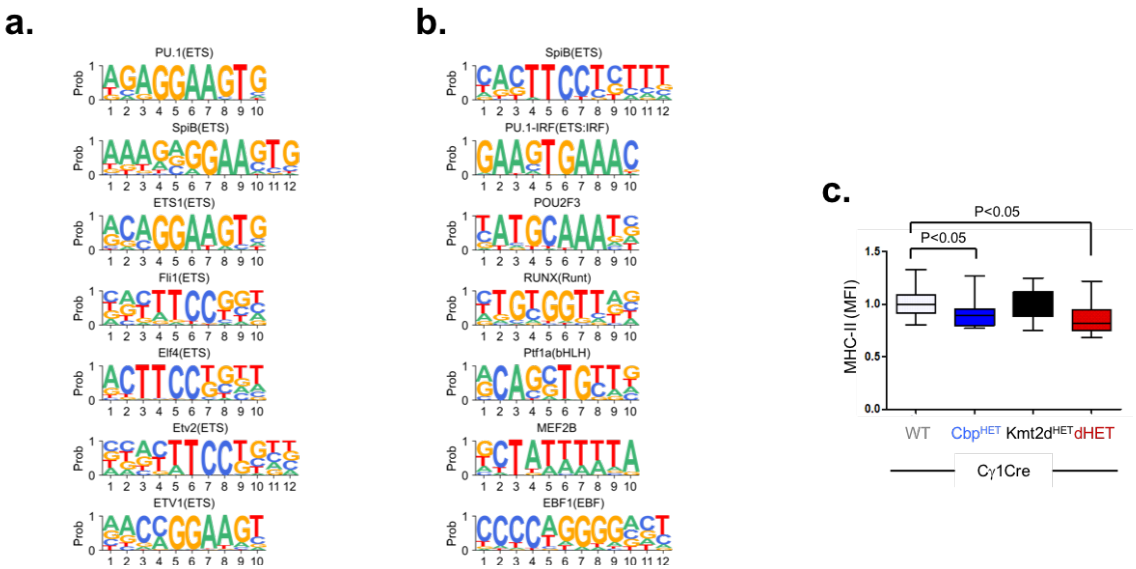

Supplementary Figure 2. CREBBP/KMT2D co-occupied regions are enriched in ETS TF binding motifs. a. Top significantly enriched known transcription factor binding motifs identified in CREBBP/KMT2D co-bound regions by HOMER (http://homer.ucsd.edu/homer/). **b.** Top significantly enriched *de novo* transcription factor binding motifs identified in CREBBP/KMT2D co-bound regions by HOMER. **c.** Relative differences in MHC-II expression in GC B cells from the indicated genotypes, analyzed by FACS (mean ± SD; n = 3-5 mice/genotype from 2 independent experiments).

Figure S3

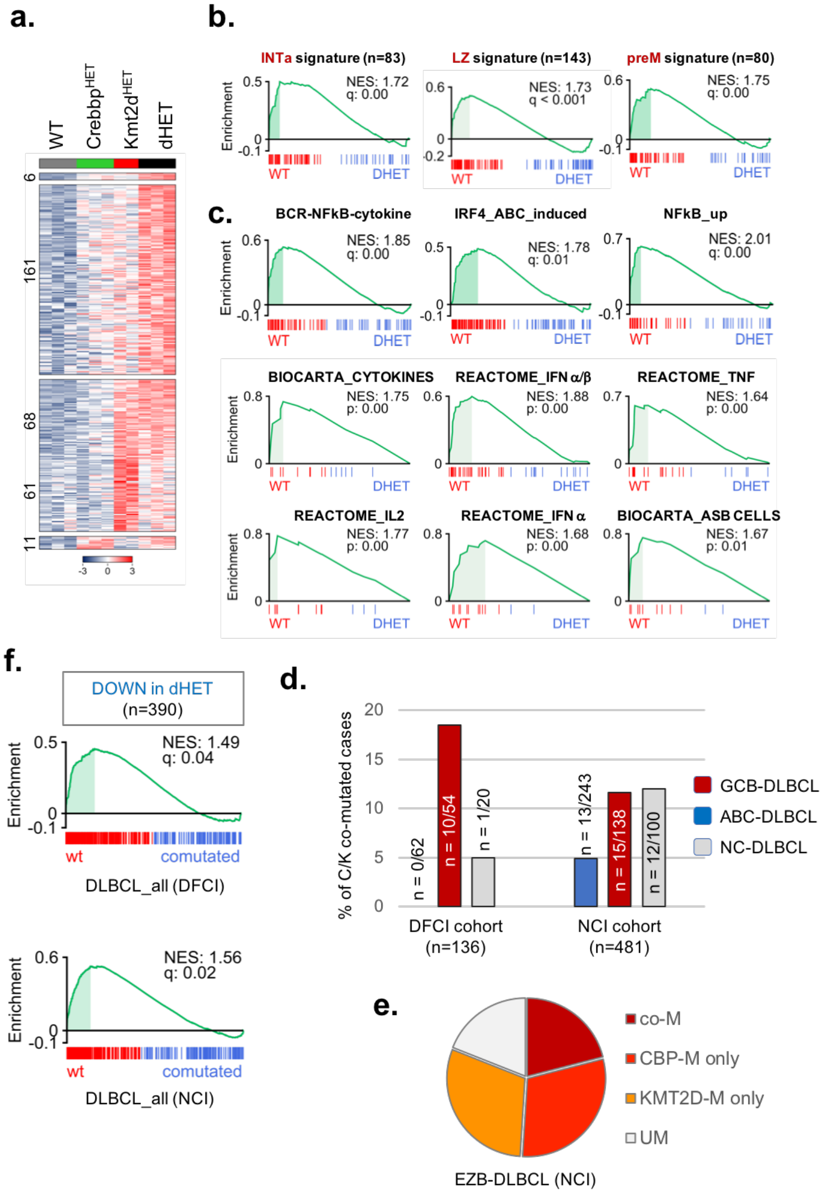

Supplementary Figure 3. Differentially expressed programs in *Crebbp/Kmt2d* dHET GC B cells and CREBBP/KMT2D co-mutated human DLBCL. a. Genes upregulated in *Crebbp*^{HET}, *Kmt2d*^{HET}, and/or dHET GC B cells, as compared to WT cells. Values on the left indicate the number of genes in each of the following categories: i) significantly upregulated in all 3 genotypes; ii) significantly upregulated in dHET cells, with trend toward increased expression in *Crebbp*^{HET} and *Kmt2d*^{HET} cells; iii) significantly upregulated in *Kmt2d*^{HET}, but not in *Crebbp*^{HET} cells (with or without changes in dHET cells); iv) significantly upregulated in *Crebbp*^{HET} but not in *Kmt2d*^{HET} cells (with or without changes in dHET cells). **b.** Gene set enrichment plot of LZ-, intermediate, and pre-memory B cell-upregulated genes in the transcriptional signatures of WT vs dHET GC B cells (LZ signature derived from Victora et al., 2012, and analogous results were obtained when using the scRNAseq-derived LZa signature from Holmes et al., 2020). **c.** Representative enrichment plots obtained by GSEA of the SignatureDB (top panles) and MSigDB (C2)

data sets in the transcriptional signatures of WT vs dHET mouse GC B cells. **d.** Proportion of CREBBP/KMT2D co-mutated cases in DLBCL phenotypic subtypes. **e.** Proportion of CREBBP/KMT2D co-mutated cases in the EZB-DLBCL genetic class. **f.** Gene set enrichment plots of dHET-downregulated genes in human DLBCLs, contrasting WT and CREBBP/KMT2D co-mutated cases (NCI cohort: 269 WT and 40 co-mutated samples, out of 481 with RNA-seq data; DFCI cohort: 92 WT and 11 co-mutated samples, out of 136 with available Affymetrix expression data. Cases harboring mutations in only one of the two genes are excluded). Note that, in this analysis, enrichment may reflect in part the segregation of CREBBP/KMT2D mutations with the GCB phenotypic subset. See Dataset S04 for full details.

Figure S4

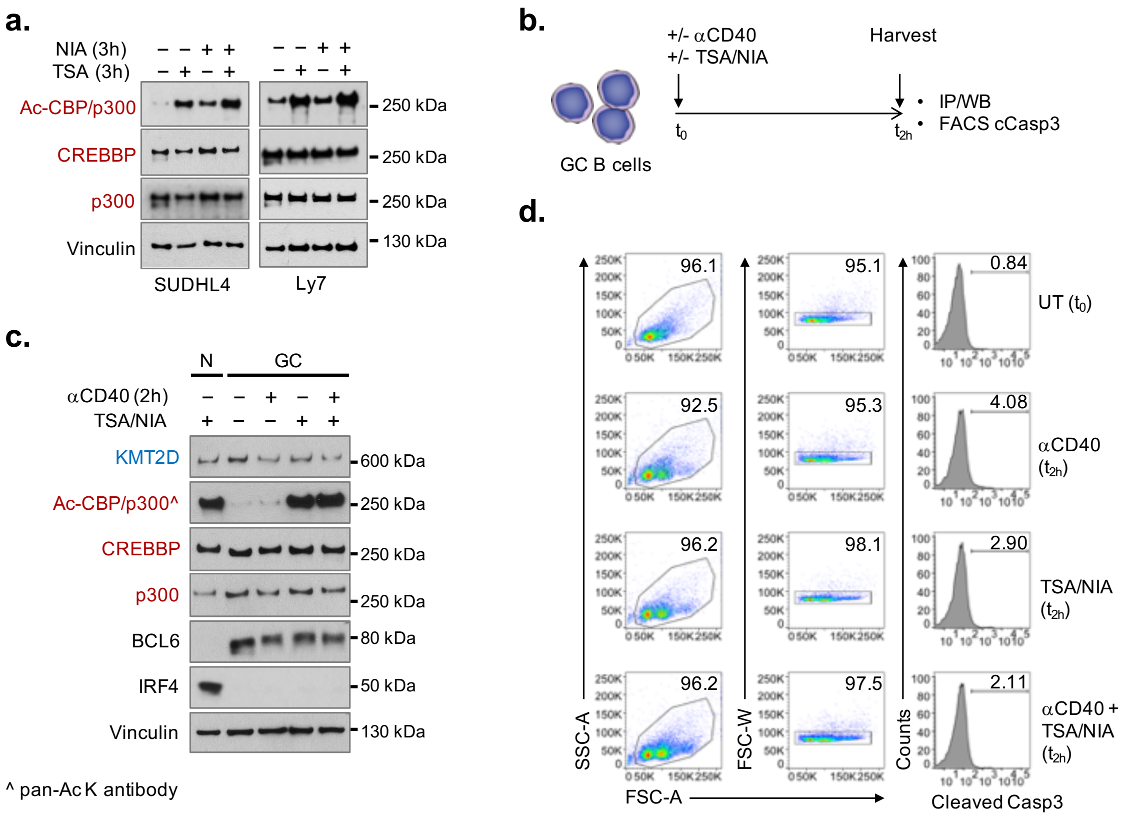

Supplementary Figure 4. Experimental approach for the analysis of KMT2D acetylation in human GC-derived B cells. a. Immunoblot analysis of the indicated proteins in whole cell extracts of SUDHL4 and LY7, treated with TSA and/or NIA. Note the increased levels of AcCBP/p300 in the treated cells, controlling for the activity of the two HDAC inhibitors. The position and size of the MW marker is provided on the right. **b.** Schematics of the experimental approach used for analysis of KMT2D acetylation in purified human GC B cells. **c.** Immunoblot analysis of the indicated proteins in naive (N) and GC B cells cultured in the presence or absence of anti-CD40, with or without TSA/NIA. Data confirm the identity of the cell populations, based on the expression pattern of IRF4 and BCL6, and document the lack of significant differences in cells cultured without anti-CD40 at this short time point. Vinculin, loading control **d.** FACS analysis of Cleaved Caspase3 in the GC B cells shown in (**c**). Cells were sequentially gated based on FSC/SSC (to exclude debris) and FSC-W (to exclude doublets), and numbers indicate the percentage of cells in the gates. In the CleavedCasp3 histogram plot, gates were set on the positive (apoptotic) cells.

a.

b.

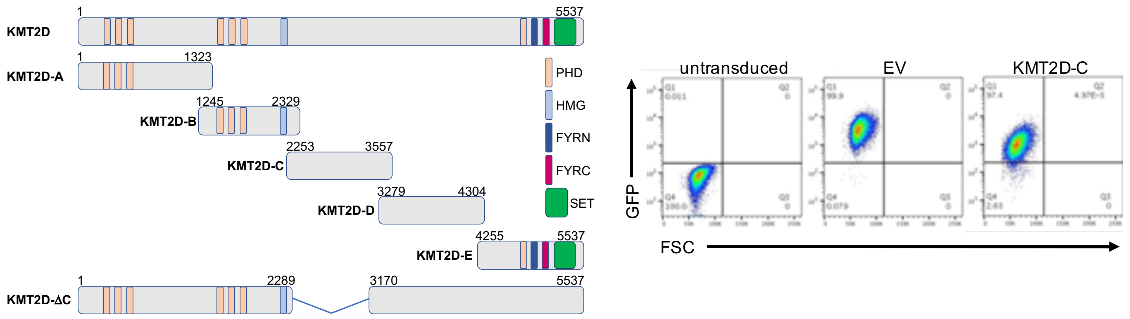

c.

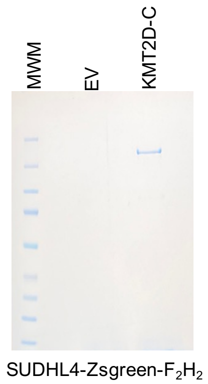

d.

Peptide Sequence	Modification	Position
KEELGASSPSYGPPNLGFVDSPSSGTHLGGLELK	1xAcetyl [K1]	K2289
TPDVFKAPLTPR	1xAcetyl [K6]	K2328
DPFAPLHKPPRPQPPEVAFK	1xAcetyl [K8]	K2468
SGITSPVEKR	1xAcetyl [K9]	K2644
REDPGTGMGSSLATAELPGTQDPGMSGLSQTELEK	1xAcetyl [K35]	K2679
GQTPFAGTQDKSSLVGLPPSK	1xAcetyl [K11]	K2745
SSLVGLPPSKLSGPILGPGSFPSDDR	1xAcetyl [K10]	K2755
GLAVSGLPPQKPSAPPAPELNNSLHPTPHTK	1xAcetyl [K11]	K2933
KDIFNEHLR	1xAcetyl [K1]	K3063
LVESANEKAER	1xAcetyl [K8]	K3079
LASVLPEVKPK	1xAcetyl [K9]	K3119
LASVLPEVKPKVEEGGR	1xAcetyl [K11]	K3121
HPSPCQFTIATPKVEPAPAANSLGLGLKPGQSMMGSR	1xAcetyl [K13]	K3140
VEPAPAANSLGLGLKPGQSMMGSR	1xAcetyl [K15]	K3155
MGTGPFSSSGHTAEKASFGATGGPPAHLLTPSPLSGPGGSSLLEK	1xAcetyl [K15]	K3182

Supplementary Figure 5. The KMT2D acetylated region maps to amino acids 2289-3170. **a.** Diagram of the full-length KMT2D protein, with annotated functional domains and their approximate location. Five deletion mutants tiled across the protein and a full-length construct lacking amino-acids 2289-3170 (all FLAG-HA double-tagged) are aligned below. Numbering is according to NCBI accession No. NP_003473.3. **b.** FACS analysis of GFP in SUDHL4 cells sorted after transduction with a lentiviral vector expressing the tagged KMT2D-C region, or an empty vector (EV) as control. **c.** Coomassie staining of the semi-purified F_2H_2-KMT2D-C protein obtained from stably transduced SUDHL4 cells by sequential immunoprecipitation with FLAG and HA, and used for mapping acetylated lysines by LC-MS/MS. Cells were cultured with TSA/NIA for 3 hours before harvesting **d.** List of high confidence acetylated peptides identified in the KMT2D-C region by LC-MS/MS. Note that mass spectrometry also identified a few acetylated lysines in the KMT2D-E region.

Figure S6

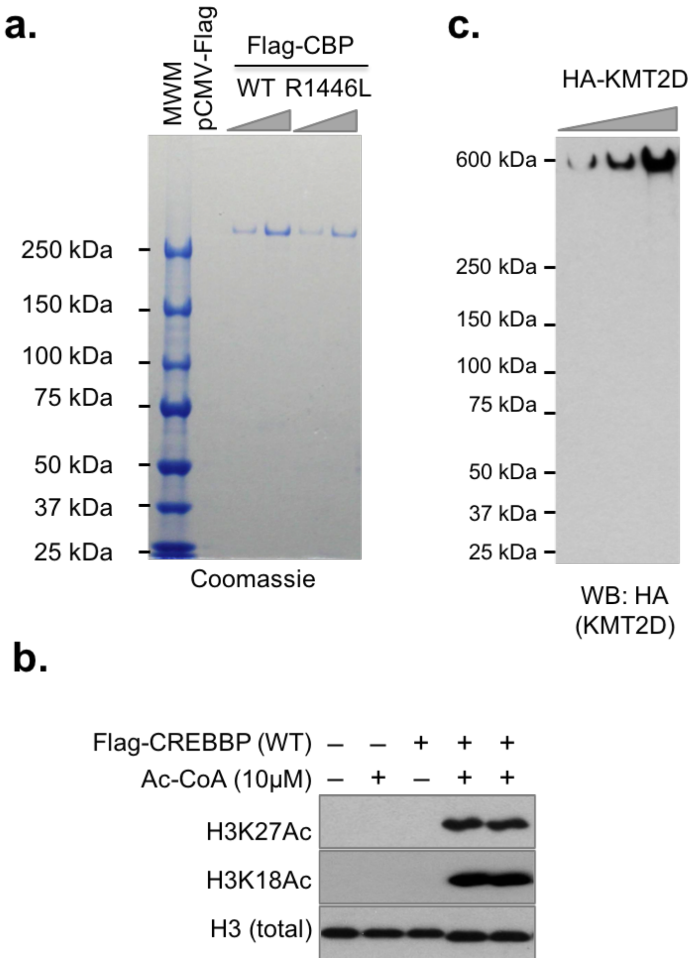

Supplementary Figure 6. Semi-purified proteins used in the *in vitro* acetylation assays. **a.** Coomassie blue stain of Flag-CREBBP proteins (WT and R1446L) obtained from transfected 293T cells by affinity immunoprecipitation and FLAG-peptide elution. **b**. Western blot analysis of H3K27Ac and H3K18Ac in *in vitro* acetylation reactions performed using the Flag-CREBBP WT protein prepared in (**a**) and recombinant nucleosomes, to document an active protein. Total H3 controls for loading (note that the same reaction was loaded in parallel on three gels to prevent stripping). **c.** Immunoblot analysis of HA-KMT2D proteins obtained from transfected 293T cells by affinity immunoprecipitation and HA-peptide elution (loading corresponds to 1/30, 1/15 and 1/7.5 of the eluate).

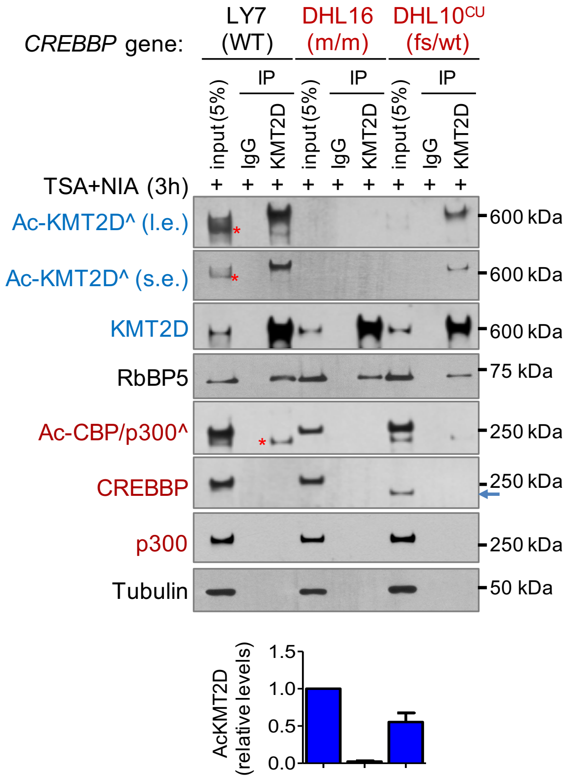

Supplementary Figure 7. KMT2D acetylation levels are reduced in CREBBP-mutated DLBCL cell lines. KMT2D acetylation levels in representative DLBCL cell lines harboring *CREBBP* or *EP300* mutations, as compared to *CREBBP*-WT cell lines. Inputs correspond to 5% of the KMT2D-IP, and IgG was used as control for the specificity of the signal. Acetylation is detected by a pan-AcK antibody (^), and asterisks denote non-specific signal detected in the inputs. Arrow points to the truncated CREBBP protein in the SUDHL10^{CU} cell line. Note that CREBBP is not detected in these small-scale IPs that were performed on whole cell extracts. In the bottom panel, relative Ac-KMT2D levels in the three cell lines are measured by densitometry after normalization for total KMT2D in the IP, with LY7 arbitrarily set at 1.

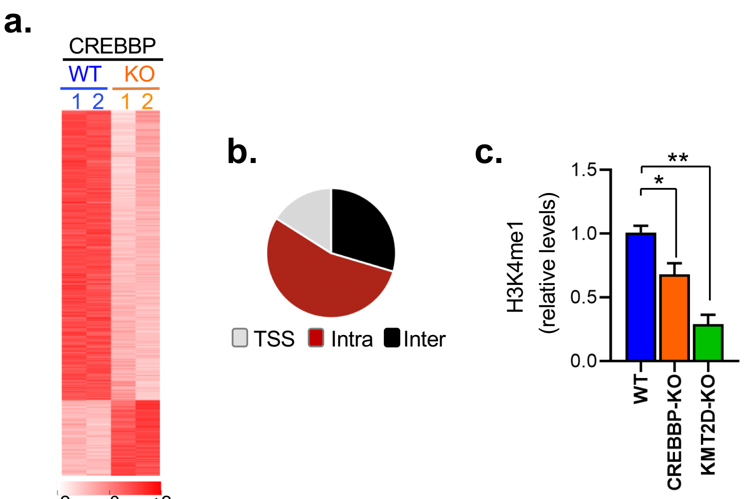

Supplementary Figure 8. ChIP-seq analysis of H3K4me1 in CREBBP-deficient DLBCL cells. a. Heatmap of H3K4me1 ChIP-seq signal in regions differentially enriched between *CREBBP*^{WT} and *CREBBP*^{KO} (AcKMT2D-impaired) SUDHL4 cells (n=2 clones each)(DESeq2, FDR<0.01, FC>1.5). Scale bar indicates the z-score. **b.** Genomic annotation of the regions shown in (**a**). **c.** H3K4me1 levels in chromatin extracts from isogenic SUDHL4 cells carrying disrupted *CREBBP* or *KMT2D* alleles, as assessed by immunoblot analysis relative to control WT cells (n=4 clones/genotype; mean ± SD, with the mean level of WT clones arbitrarily set as 1). * P < 0.005; ** P < 0.001, two-tailed Student's t-test. Quantification of band intensities was obtained by densitometry using ImageJ.
