# Supplementary material for: KMT2D acetylation by CREBBP reveals a cooperative functional interaction at enhancers in normal and malignant germinal center B cells
Source: Proc Natl Acad Sci U S A. 2023 Mar 9;120(11):e2218330120. doi: 10.1073/pnas.2218330120 (PMC10089214; doi:10.1073/pnas.2218330120)
Supplement: Supplementary file 7 — Dataset S06 (PDF) [file pnas.2218330120.sd06.pdf]

Figure 4A

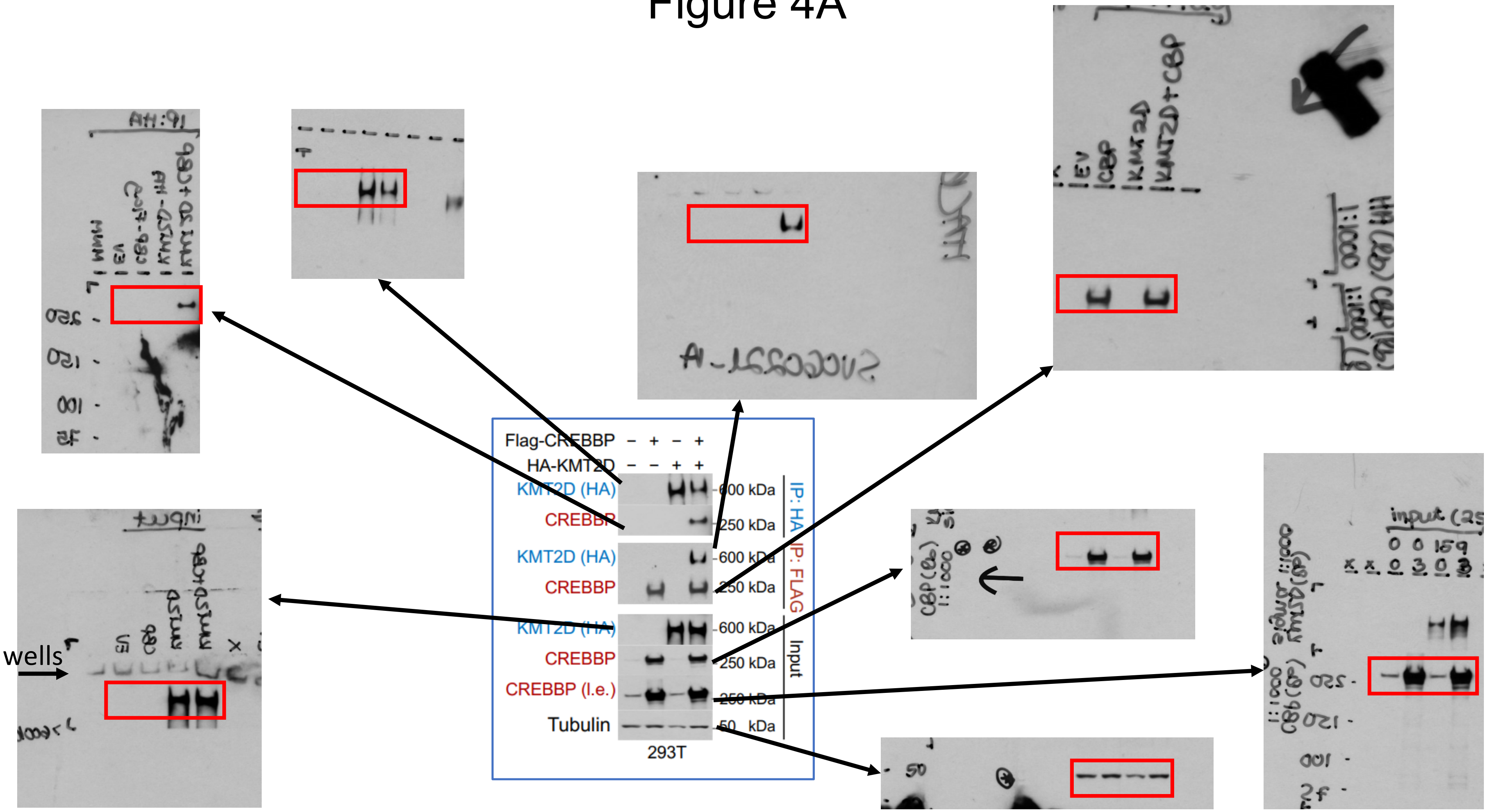
Flag-CREBBP – + – +
HA-KMT2D – – + +
KMT2D (HA)
CREBBP
KMT2D (HA)
CREBBP
KMT2D (HA)
CREBBP
CREBBP (l.e.)
Tubulin
600 kDa
250 kDa
600 kDa
250 kDa
600 kDa
250 kDa
250 kDa
50 kDa
IP: HA
IP: FLAG
Input
293T
wells

Figure 4B

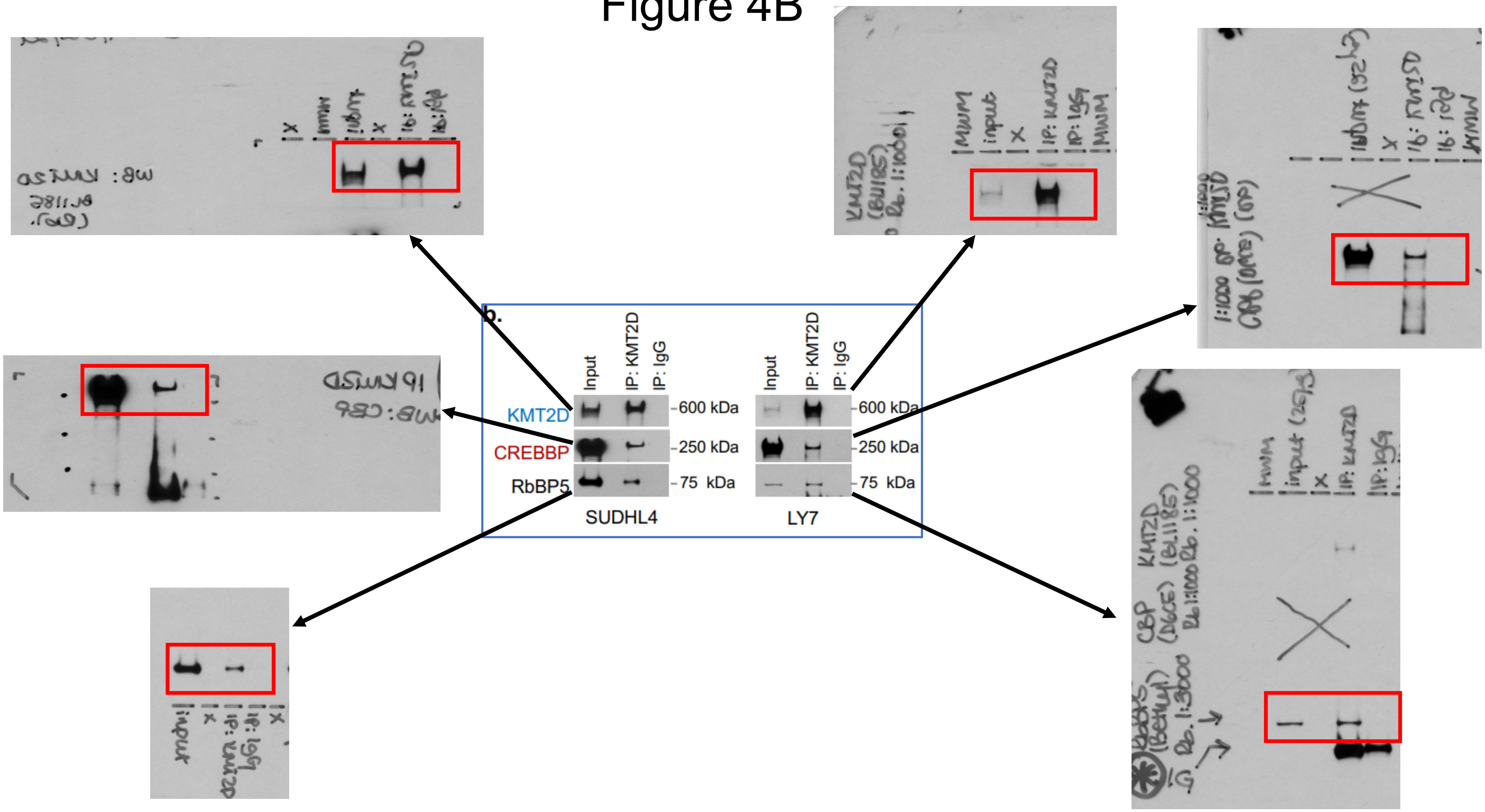
b.
Input
IP: KMT2D
IP: IgG
KMT2D
CREBBP
RbBP5
600 kDa
250 kDa
75 kDa
SUDHL4
LY7

Figure 4C

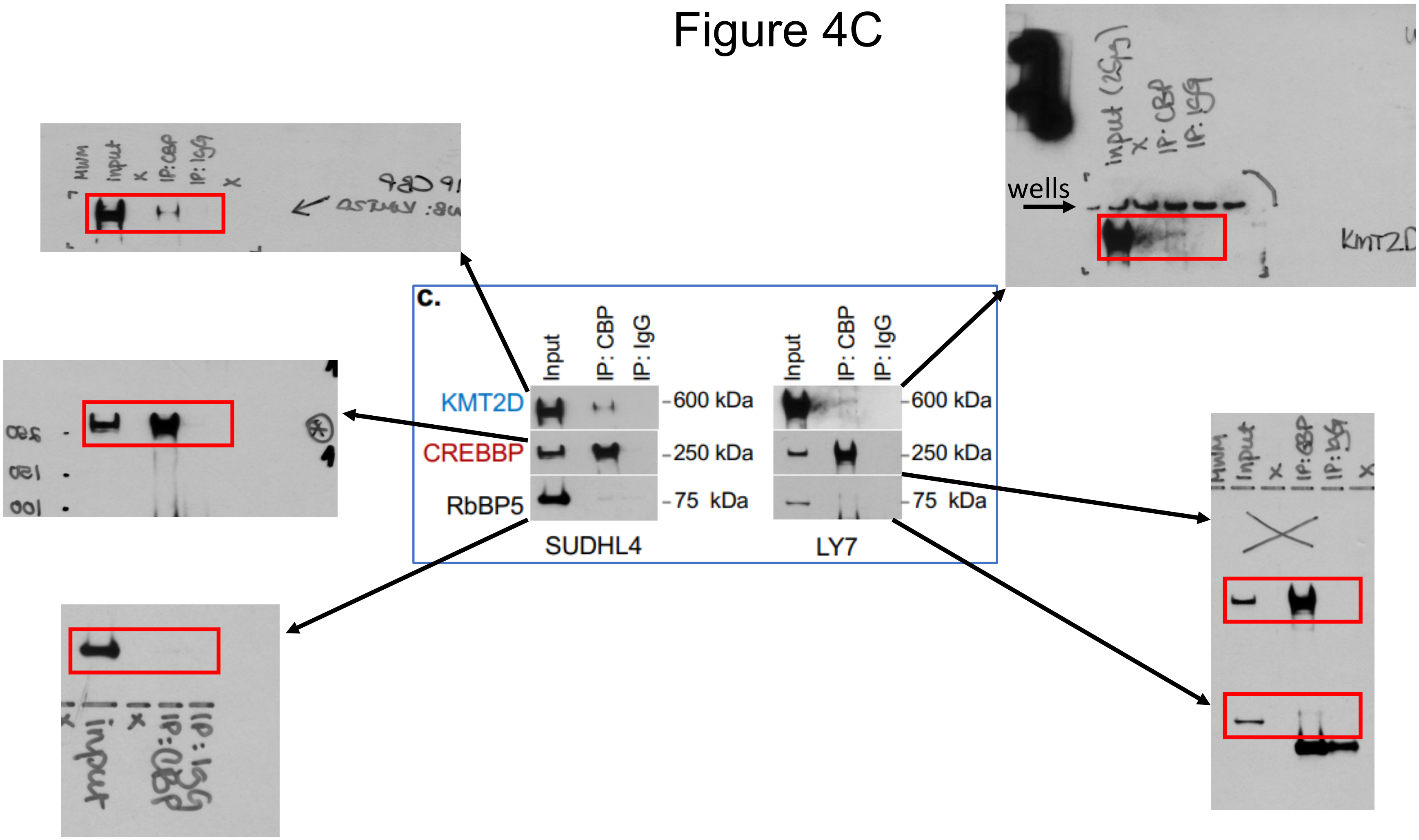
c.
Input
IP: CBP
IP: IgG
KMT2D
CREBBP
RbBP5
600 kDa
250 kDa
75 kDa
SUDHL4
LY7
wells

Figure 5B

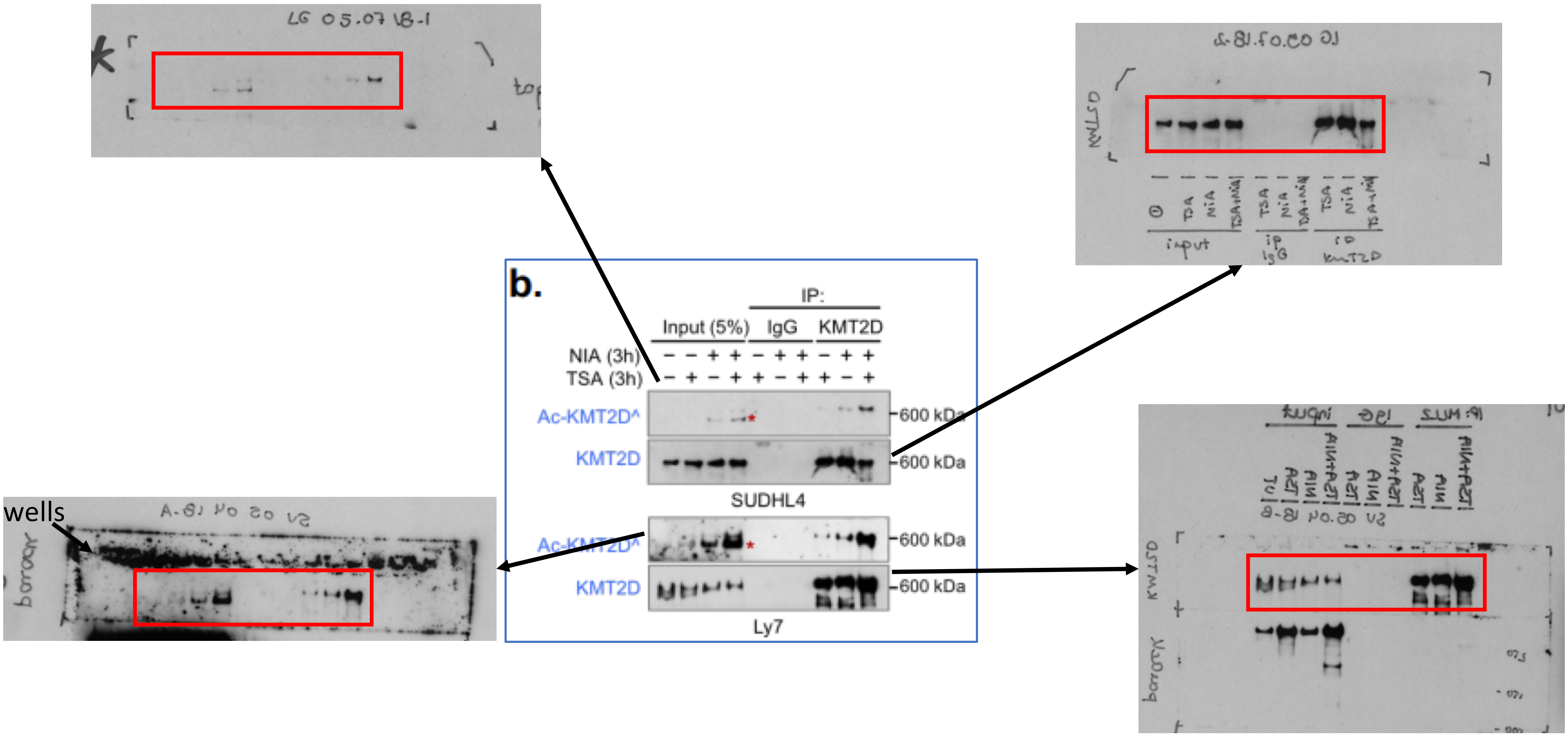

LG 05.07 IB-1
wells
b.
IP:
Input (5%)
IgG
KMT2D
NIA (3h)
TSA (3h)
Ac-KMT2D^
KMT2D
600 kDa
SUDHL4
Ly7
input
ip IgG
ip KMT2D

Figure 5C

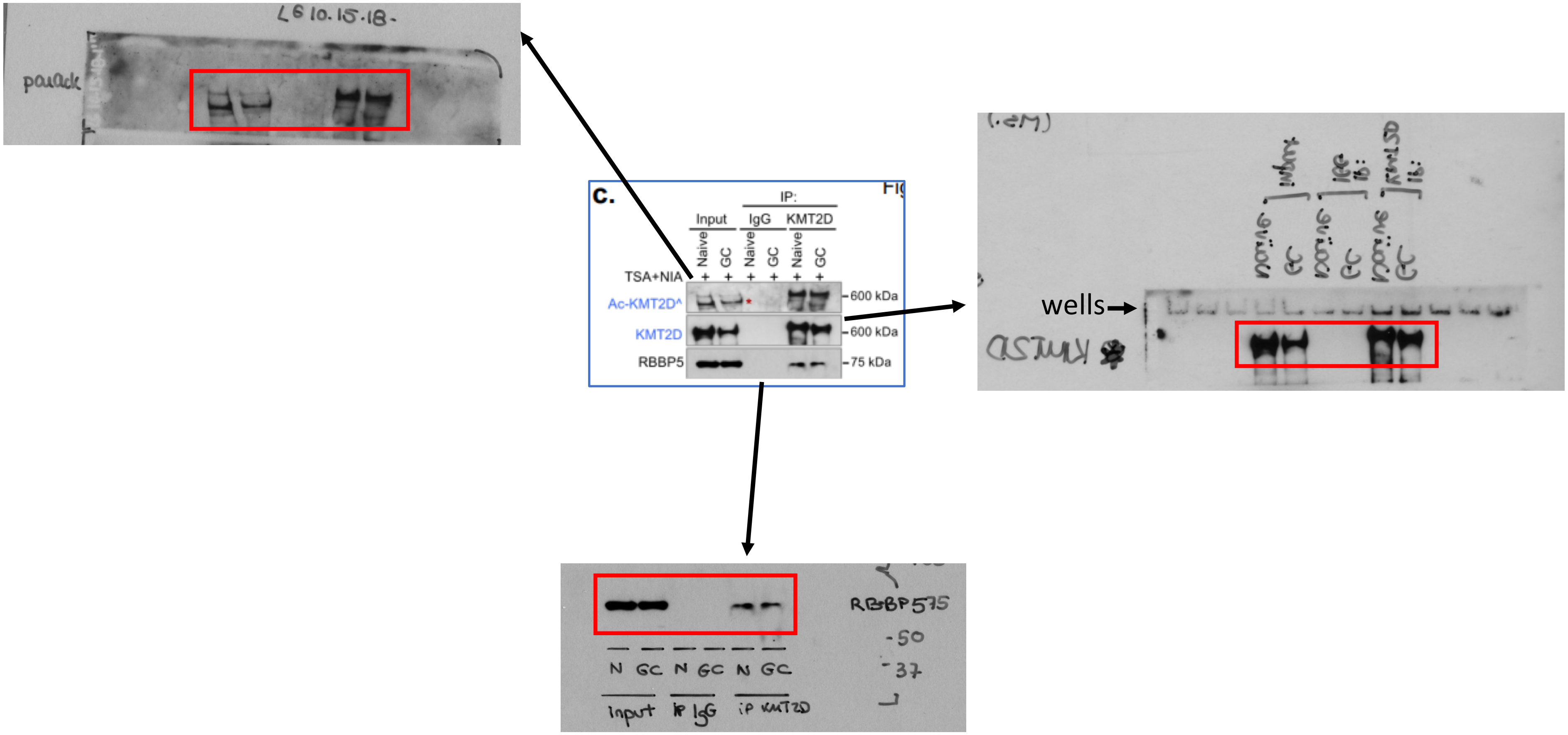

C.
IP:
Input
IgG
KMT2D
Naive
GC
TSA+NIA
Ac-KMT2D^
KMT2D
RBBP5
600 kDa
600 kDa
75 kDa
wells
RBBP5 75
-50
-37
N GC N GC N GC
Input
IP IgG
IP KMT2D

Figure 5D

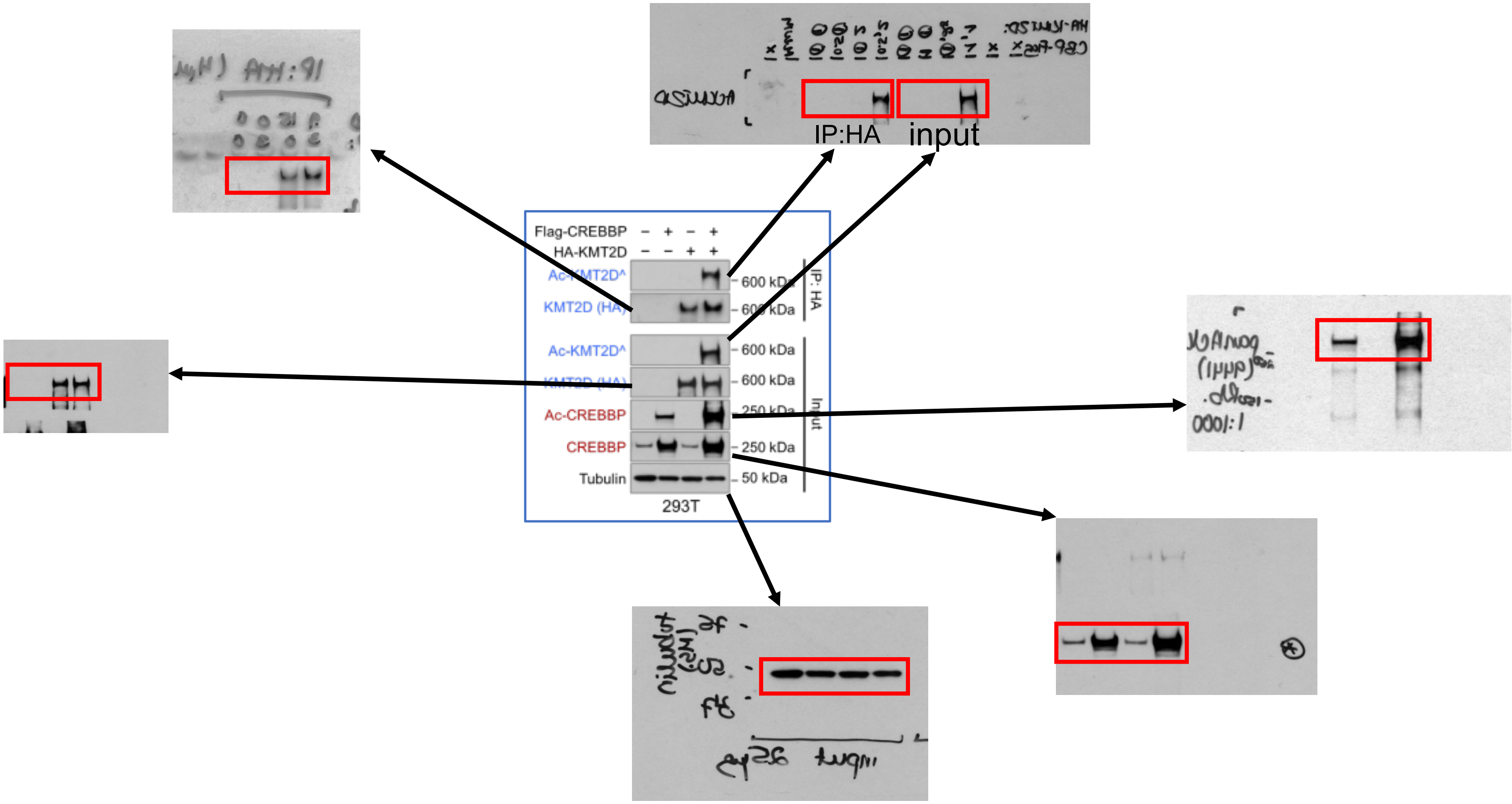

IP:HA
input
Flag-CREBBP – + – +
HA-KMT2D – – + +
Ac-KMT2D^
KMT2D (HA)
600 kDa
600 kDa
IP: HA
Ac-KMT2D^
KMT2D (HA)
Ac-CREBBP
CREBBP
Tubulin
600 kDa
600 kDa
250 kDa
250 kDa
50 kDa
Input
293T

Figure 5E

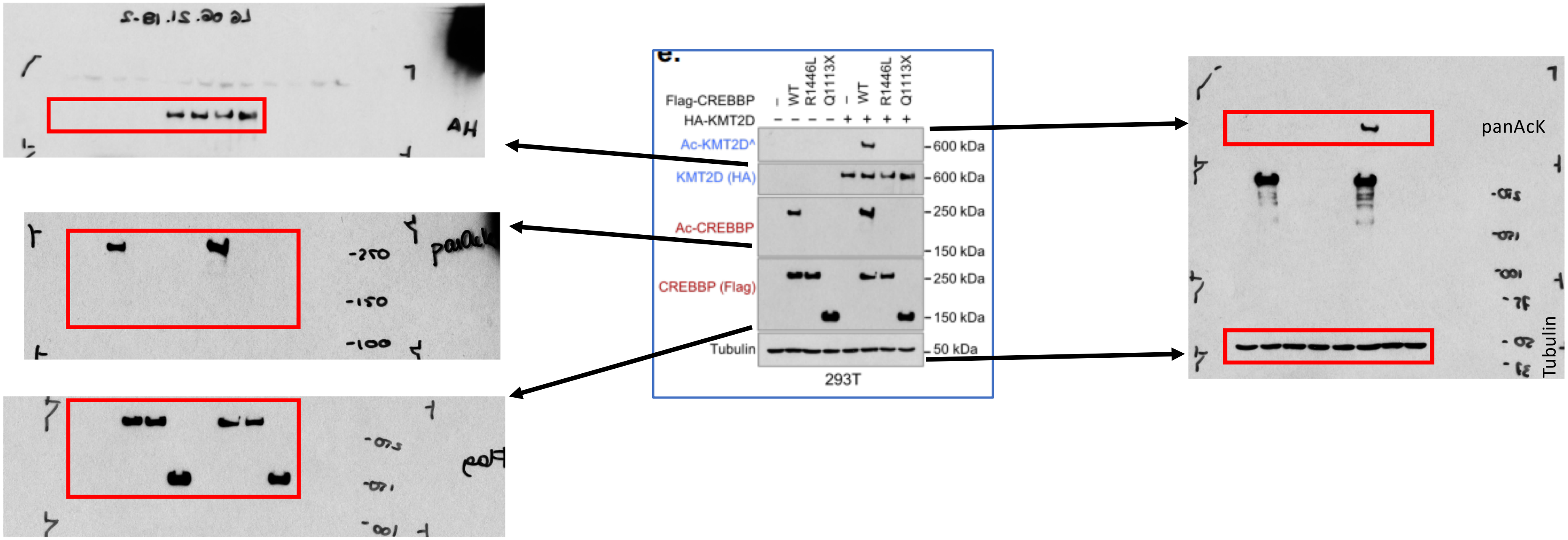

e.
Flag-CREBBP
HA-KMT2D
–
WT
R1446L
Q1113X
Ac-KMT2D^
KMT2D (HA)
Ac-CREBBP
CREBBP (Flag)
Tubulin
600 kDa
600 kDa
250 kDa
150 kDa
250 kDa
150 kDa
50 kDa
293T
panAcK
Tubulin

Figure 5F

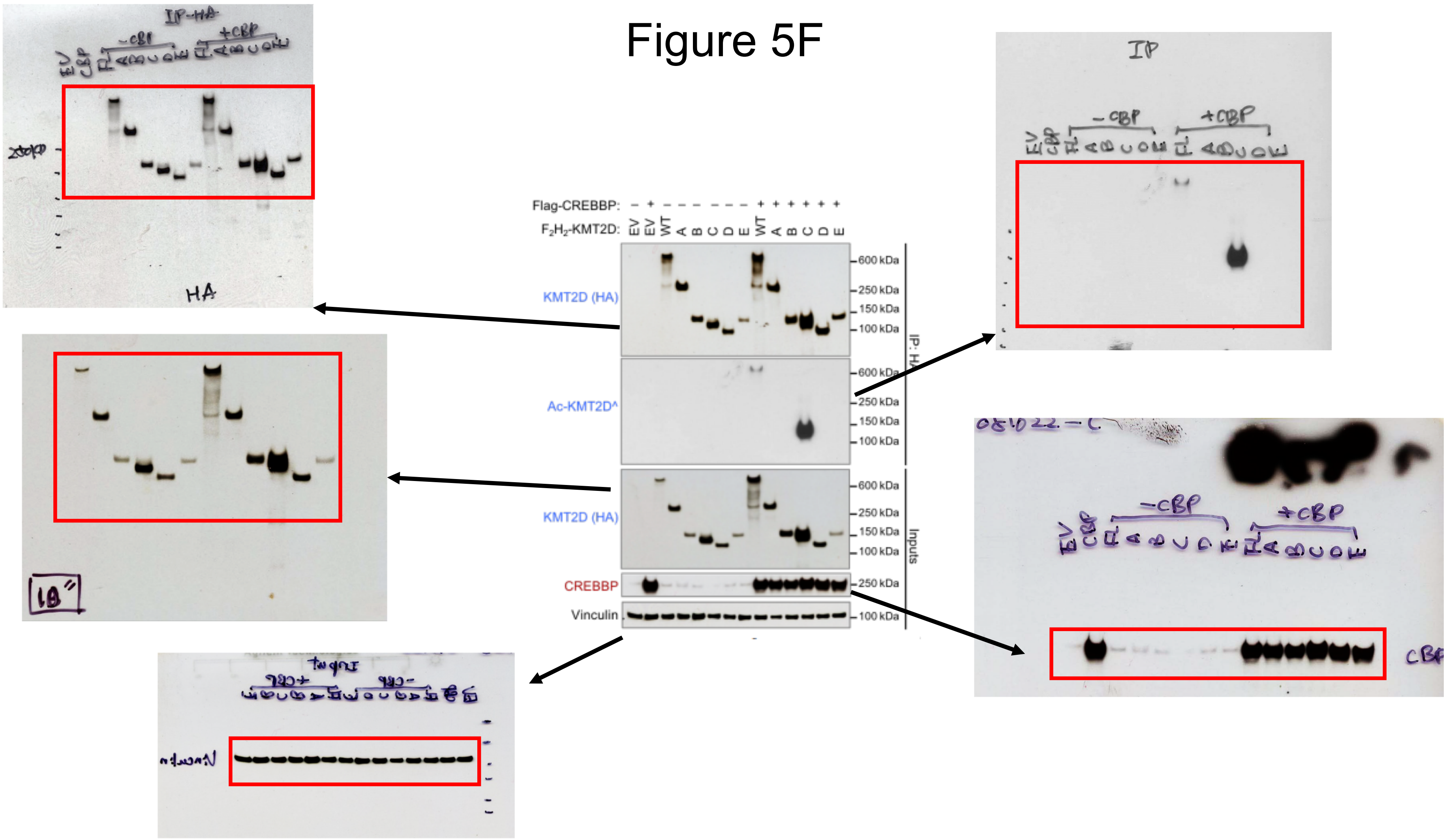

IP-HA
-CBP
+CBP
HA
Flag-CREBBP:
F$_2$H$_2$-KMT2D:
EV
WT
A
B
C
D
E
KMT2D (HA)
Ac-KMT2D^
CREBBP
Vinculin
600 kDa
250 kDa
150 kDa
100 kDa
IP: HA
Inputs
IP
IB

Figure 5G

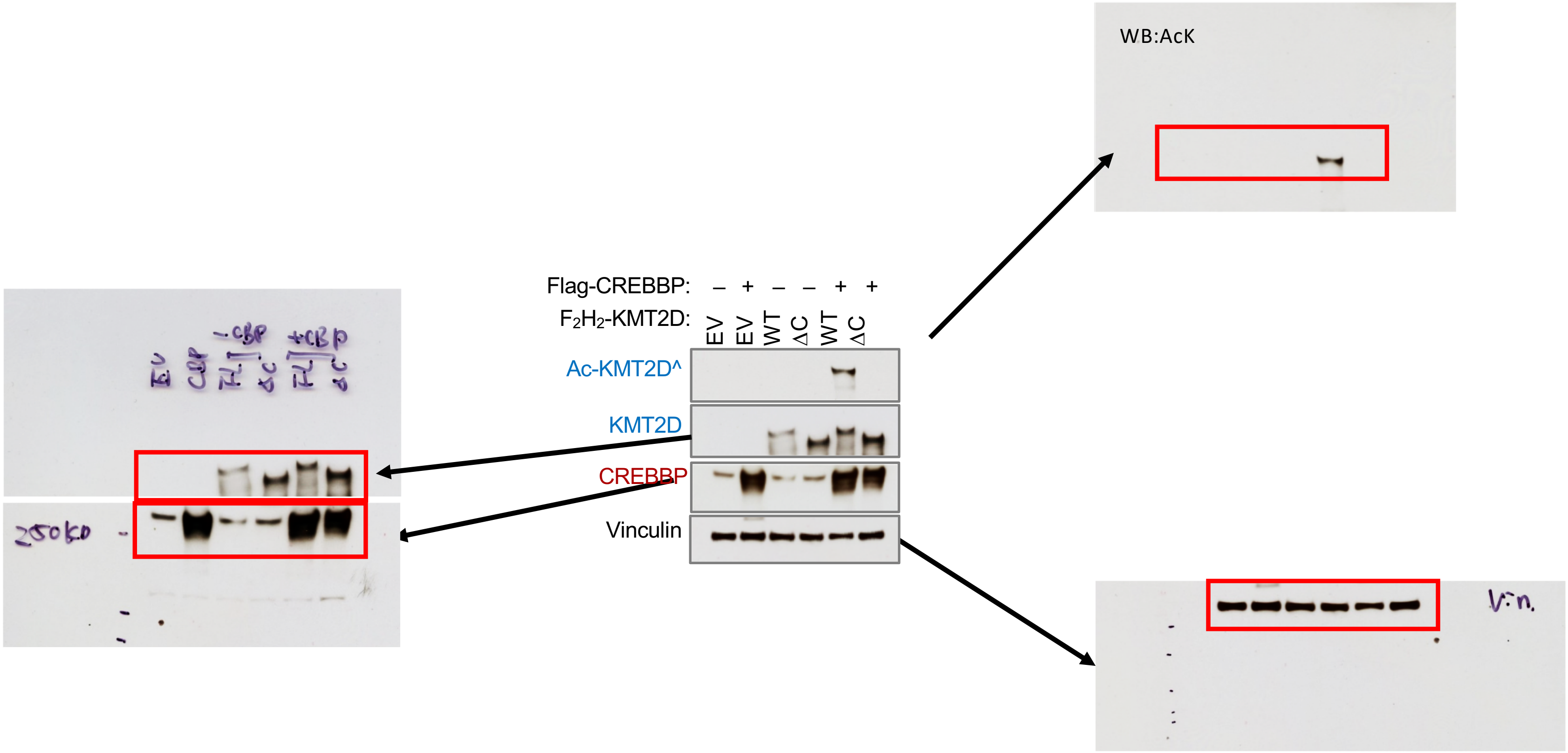

WB:AcK
Flag-CREBBP: – + – – + +
F2H2-KMT2D: EV EV WT ΔC WT ΔC
Ac-KMT2D^
KMT2D
CREBBP
Vinculin

Figure 5H

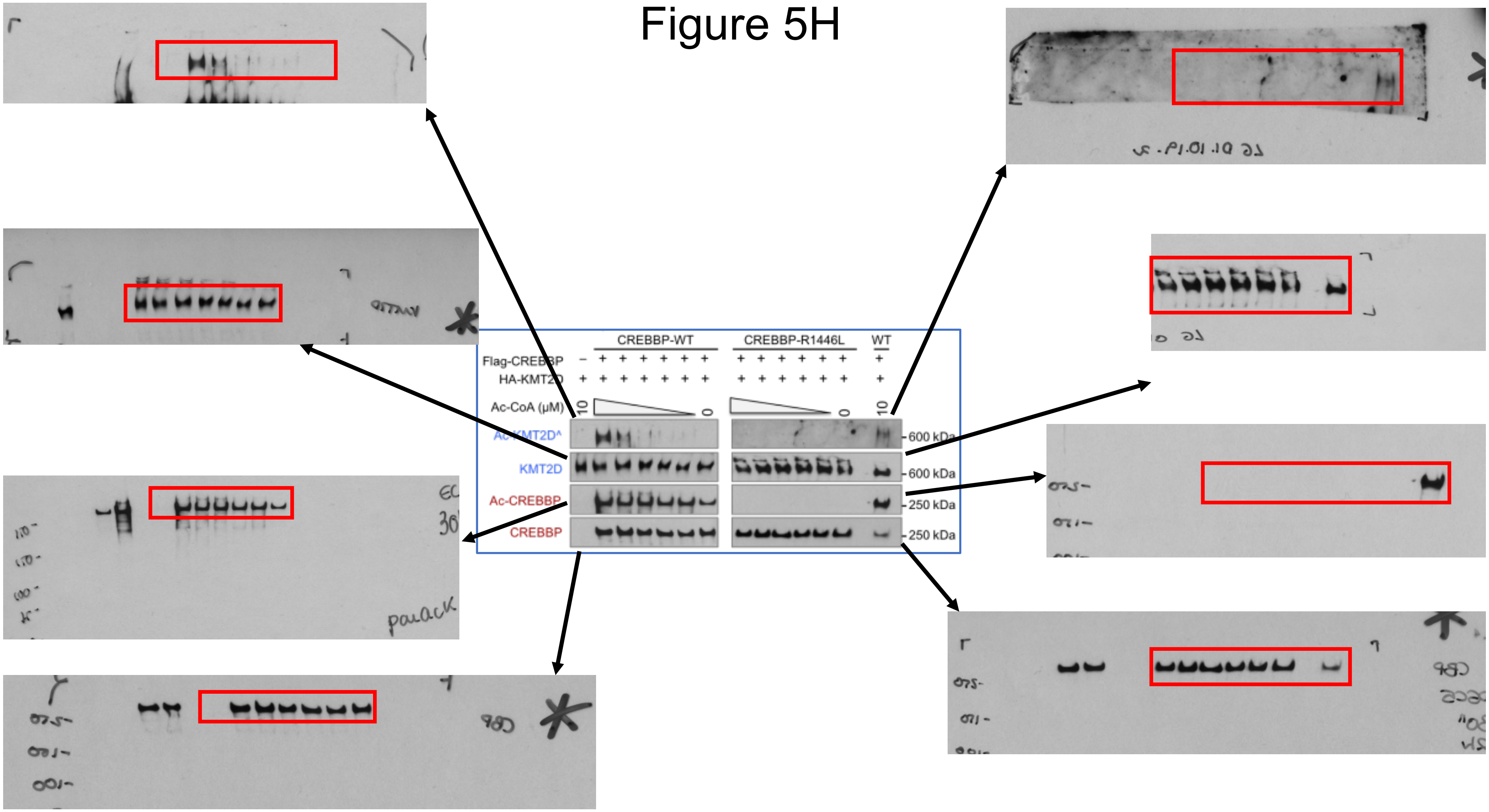

CREBBP-WT
CREBBP-R1446L
WT
Flag-CREBBP
HA-KMT2D
Ac-CoA (μM)
10
0
0
10
Ac-KMT2D^
KMT2D
Ac-CREBBP
CREBBP
600 kDa
600 kDa
250 kDa
250 kDa

Figure 6A

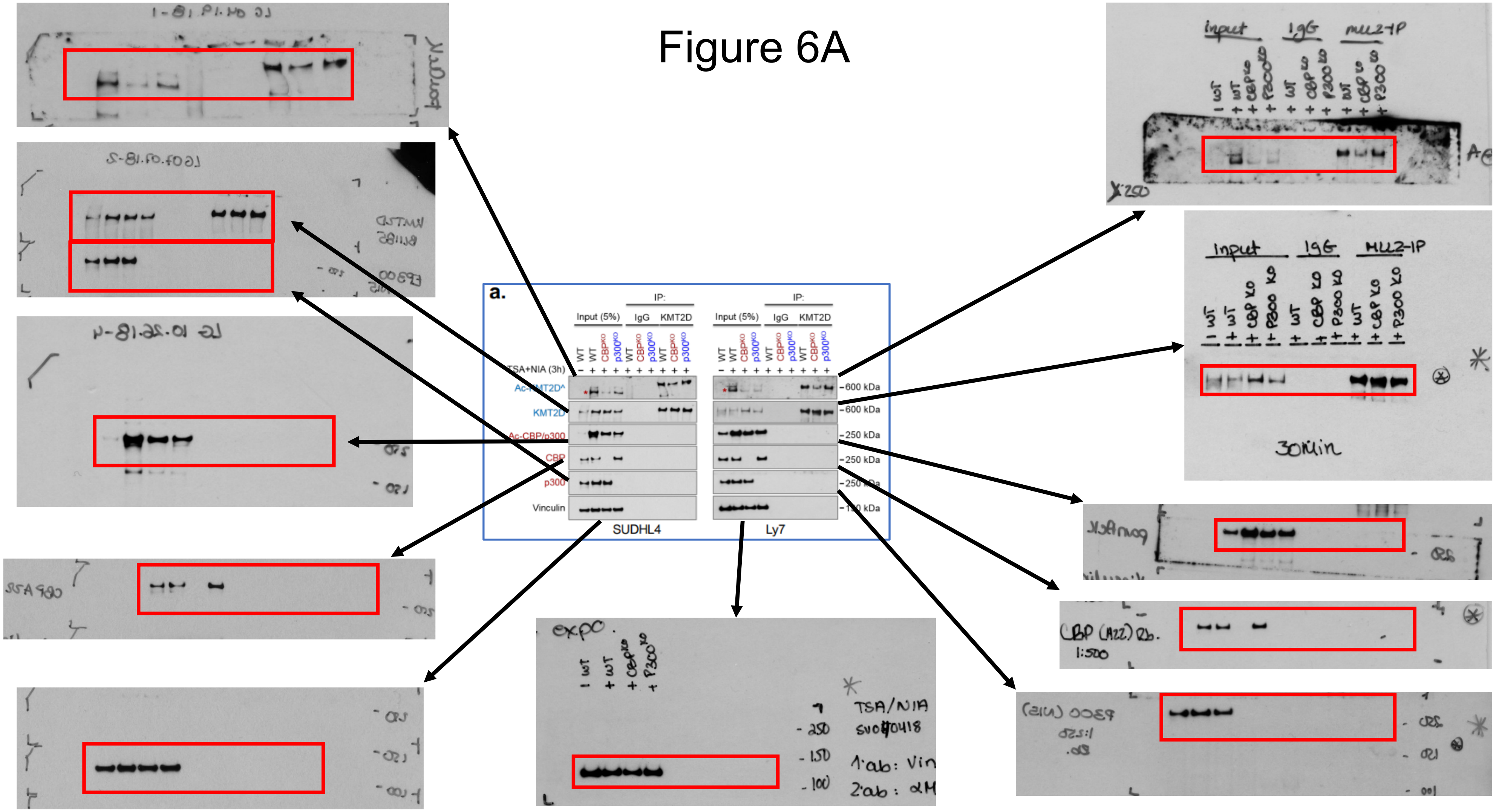
a.
IP:
Input (5%)
IgG
KMT2D
WT
CBPKO
p300KO
TSA+NIA (3h)
Ac-KMT2D
KMT2D
Ac-CBP/p300
CBP
p300
Vinculin
600 kDa
250 kDa
150 kDa
SUDHL4
Ly7
Input
IgG
MLL2-IP
30min
TSA/NIA

Figure 6C

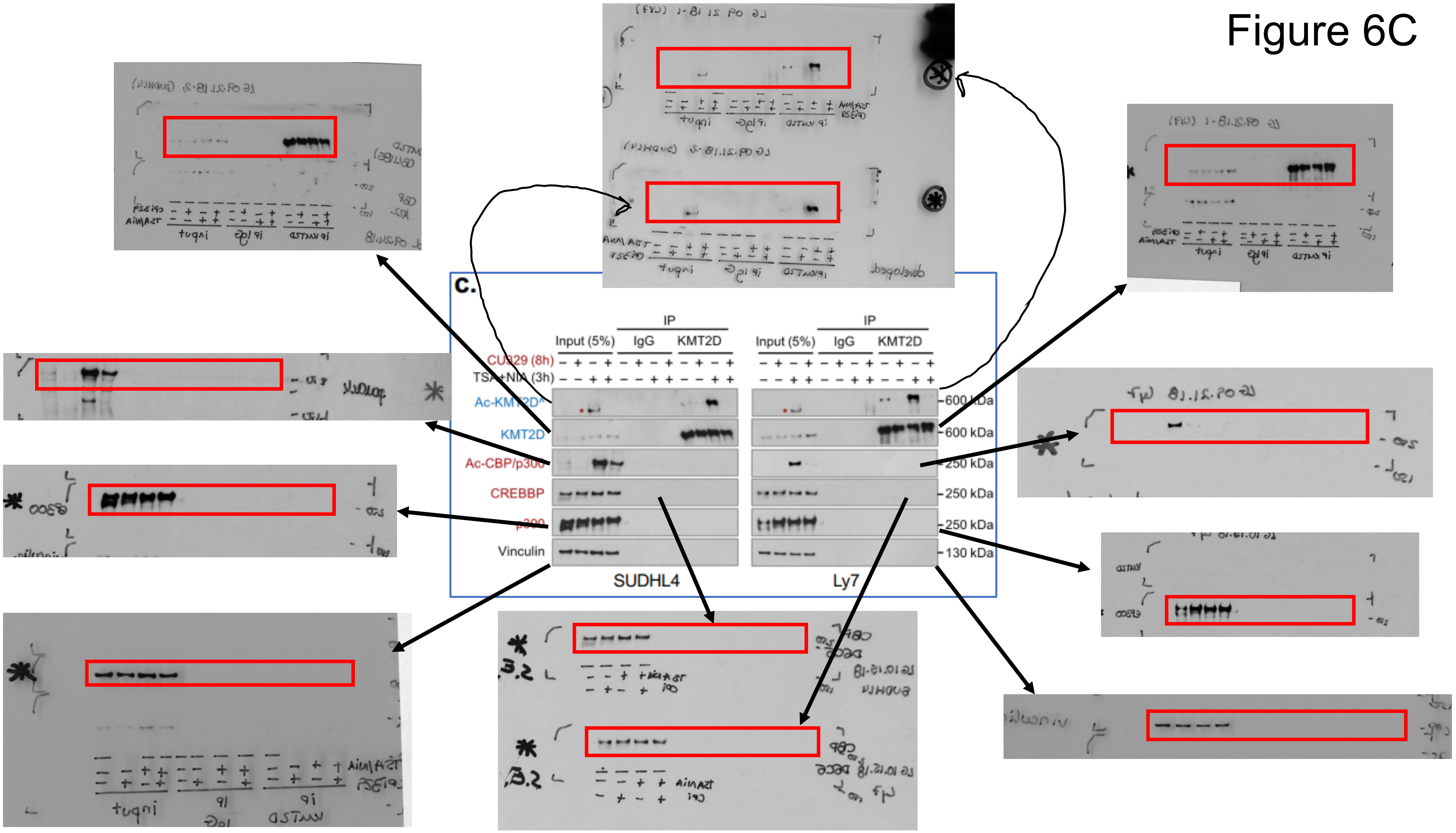

c.
IP
Input (5%)
IgG
KMT2D
TSA+NIA (3h)
Ac-KMT2D^
KMT2D
Ac-CBP/p300
CREBBP
p300
Vinculin
600 kDa
600 kDa
250 kDa
250 kDa
250 kDa
130 kDa
SUDHL4
Ly7

Figure 7B

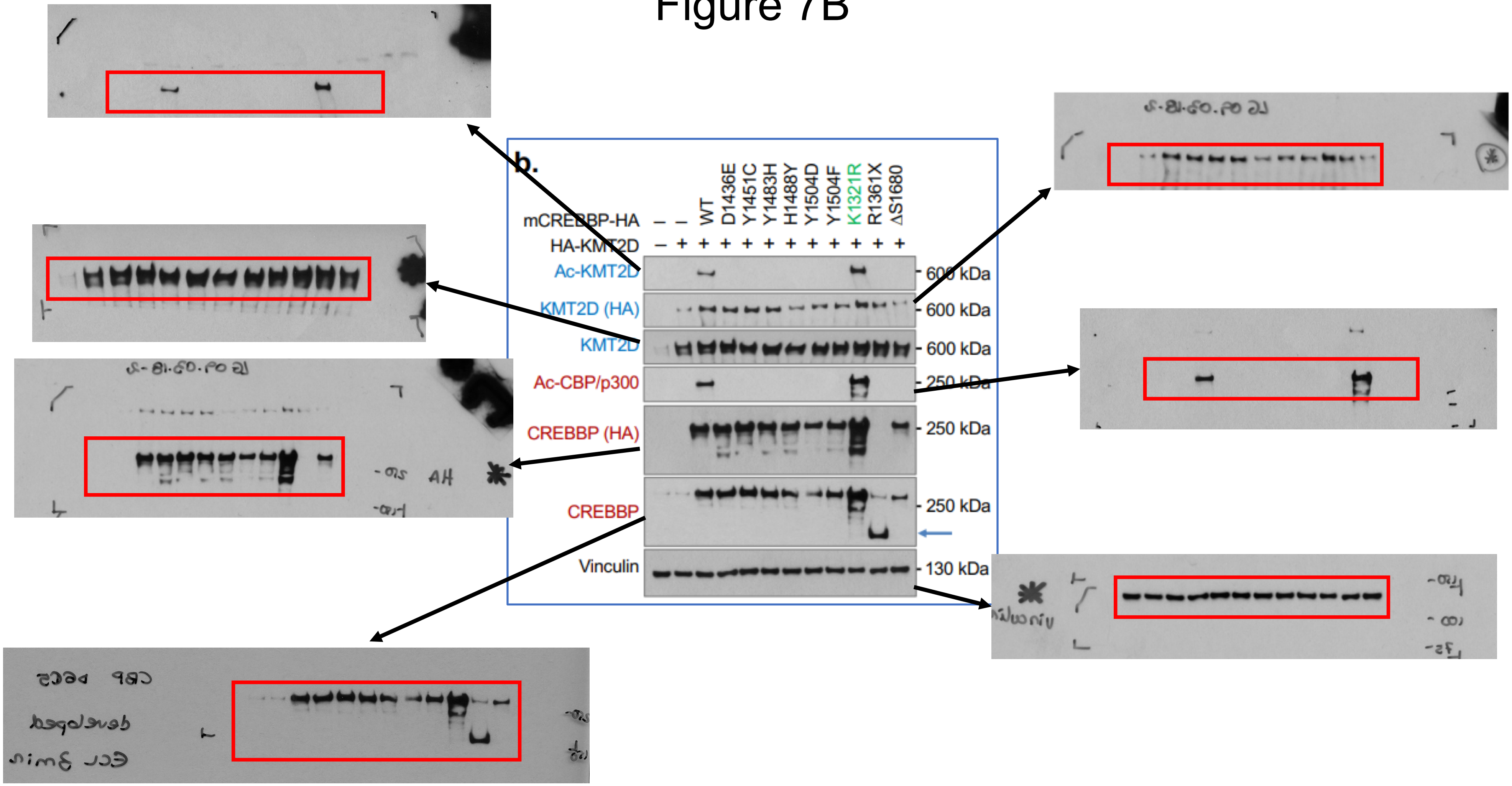

b.
mCREBBP-HA
HA-KMT2D
WT
D1436E
Y1451C
Y1483H
H1488Y
Y1504D
Y1504F
K1321R
R1361X
ΔS1680
Ac-KMT2D
KMT2D (HA)
KMT2D
Ac-CBP/p300
CREBBP (HA)
CREBBP
Vinculin
600 kDa
600 kDa
600 kDa
250 kDa
250 kDa
250 kDa
130 kDa

Figure 7C

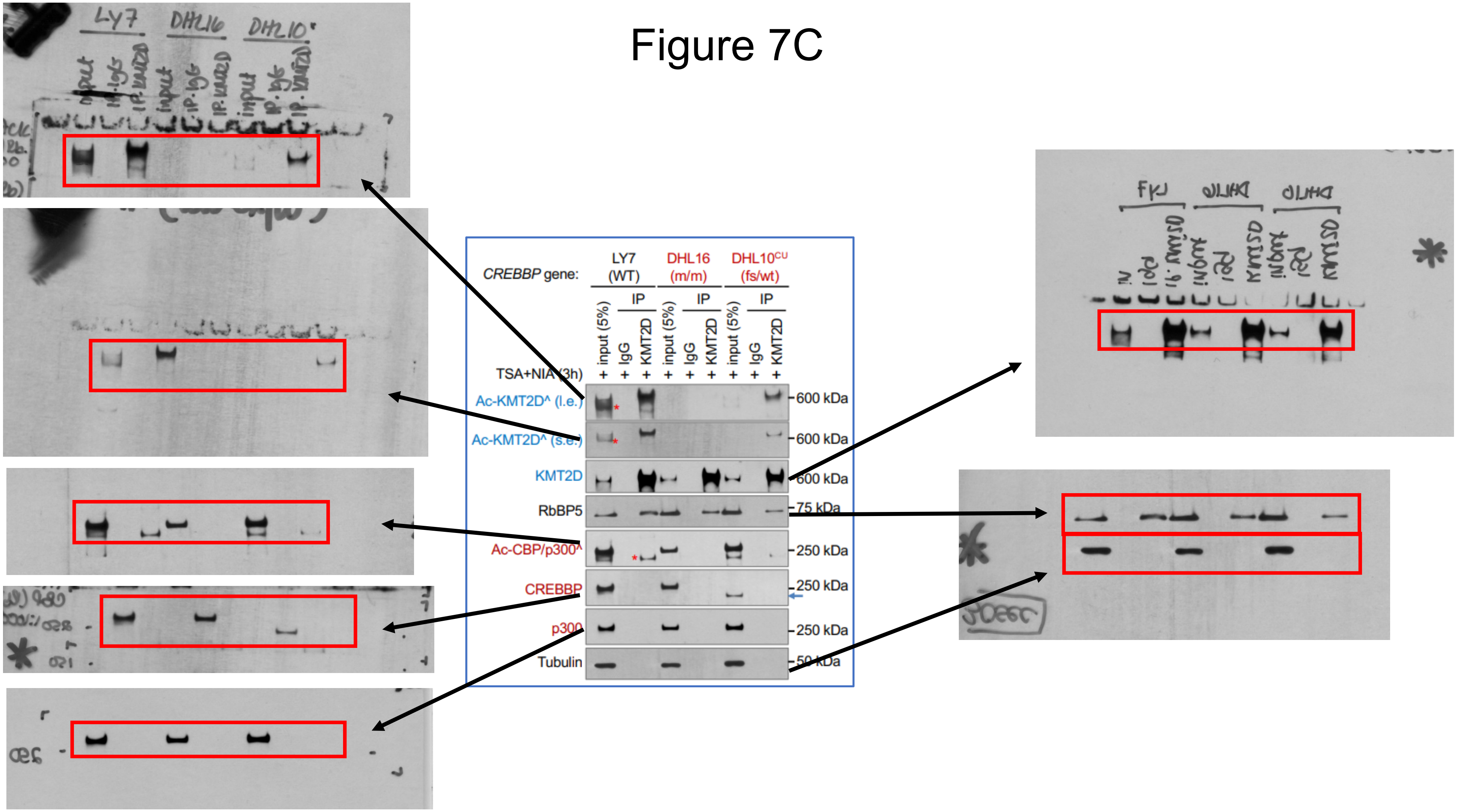

CREBBP gene:
LY7 (WT)
DHL16 (m/m)
DHL10CU (fs/wt)
IP
input (5%)
IgG
KMT2D
TSA+NIA (3h)
Ac-KMT2D^ (l.e.)
Ac-KMT2D^ (s.e.)
KMT2D
RbBP5
Ac-CBP/p300^
CREBBP
p300
Tubulin
600 kDa
600 kDa
600 kDa
75 kDa
250 kDa
250 kDa
250 kDa
50 kDa

Figure 8A

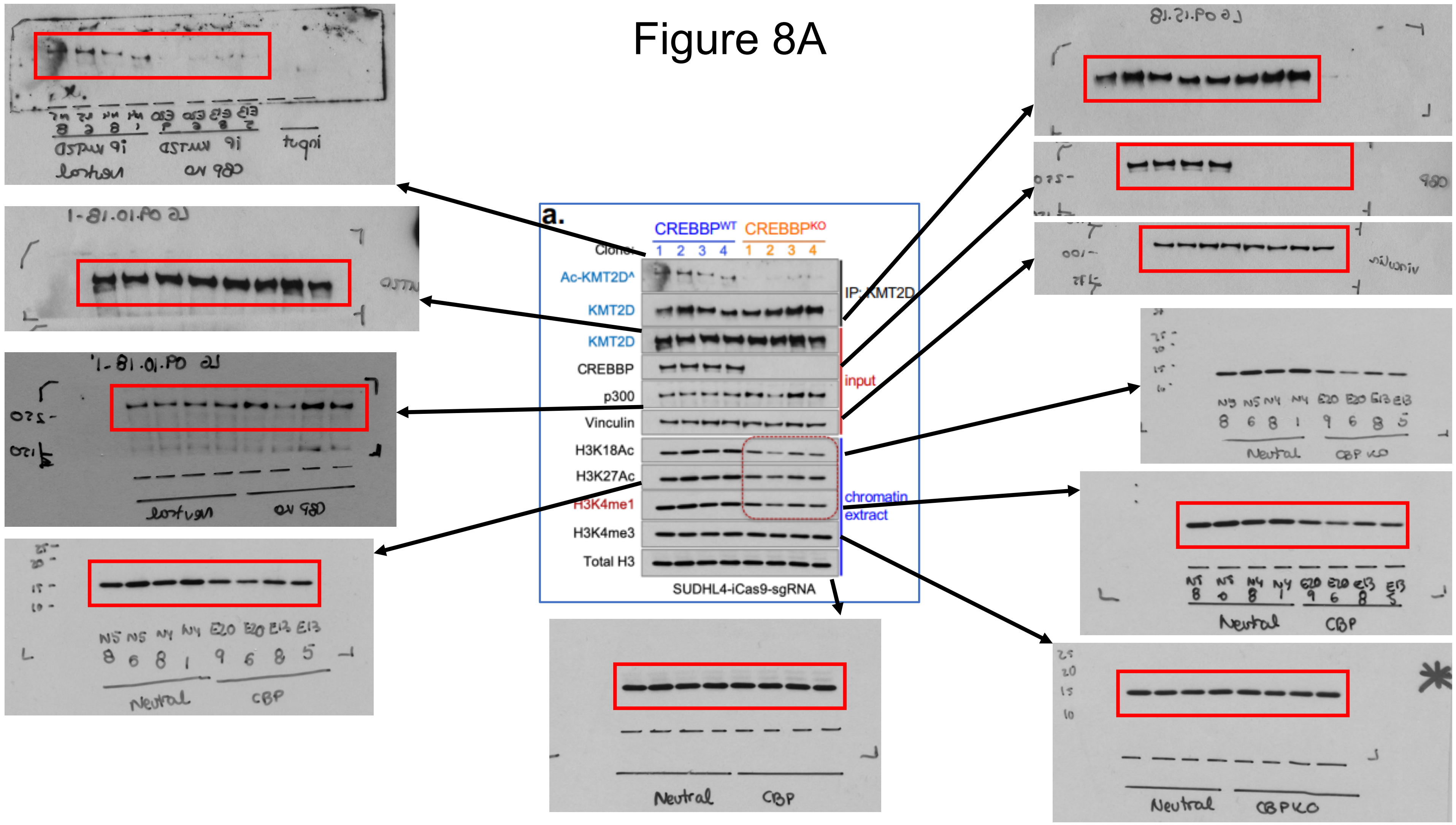
a.
CREBBP^WT
CREBBP^KO
Clone:
1 2 3 4 1 2 3 4
Ac-KMT2D^
KMT2D
KMT2D
CREBBP
p300
Vinculin
H3K18Ac
H3K27Ac
H3K4me1
H3K4me3
Total H3
IP: KMT2D
input
chromatin extract
SUDHL4-iCas9-sgRNA
Neutral
CBP
CBP KO

Figure 8C

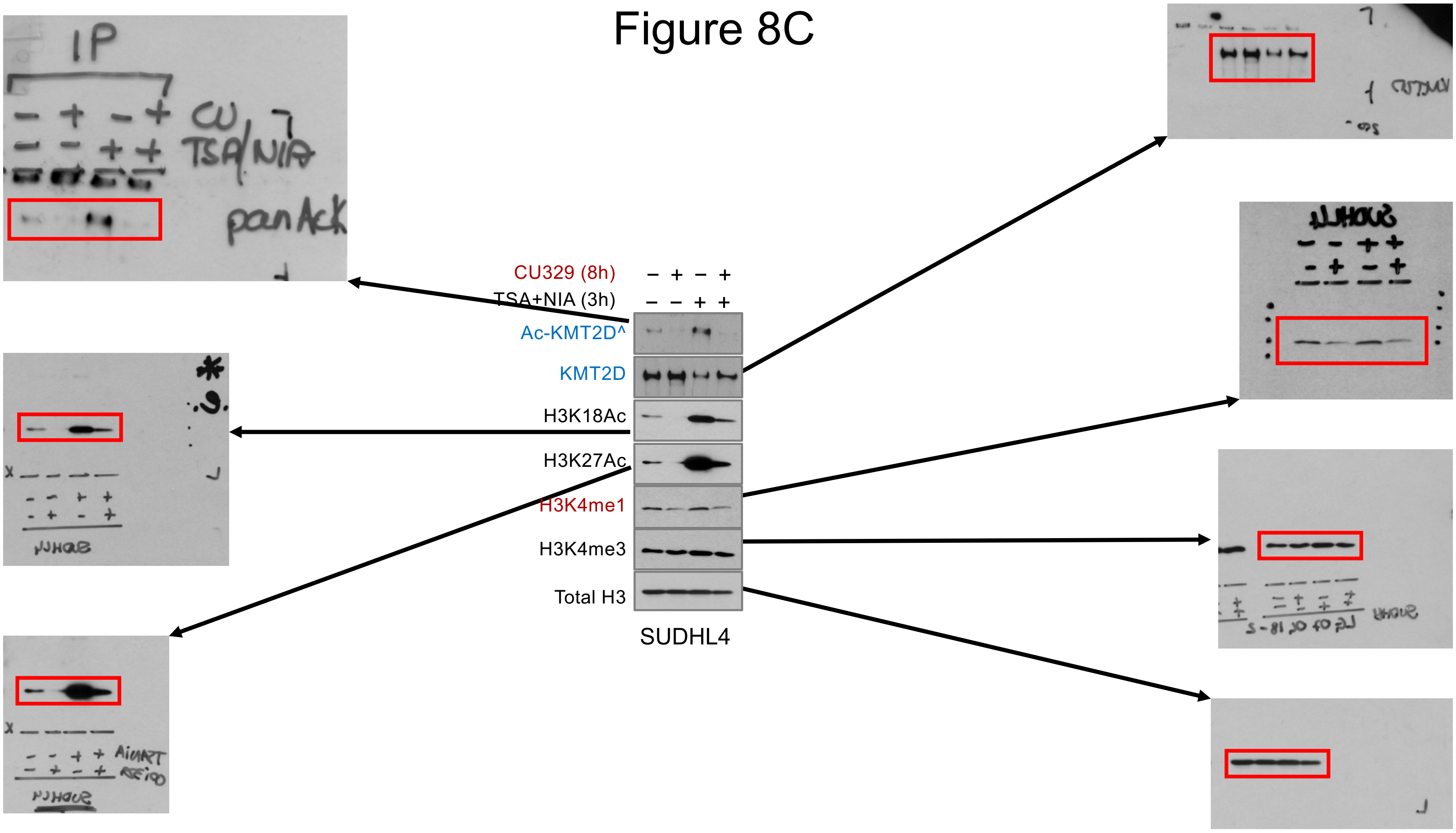
CU329 (8h)
TSA+NIA (3h)
Ac-KMT2D^
KMT2D
H3K18Ac
H3K27Ac
H3K4me1
H3K4me3
Total H3
SUDHL4

Figure S4A

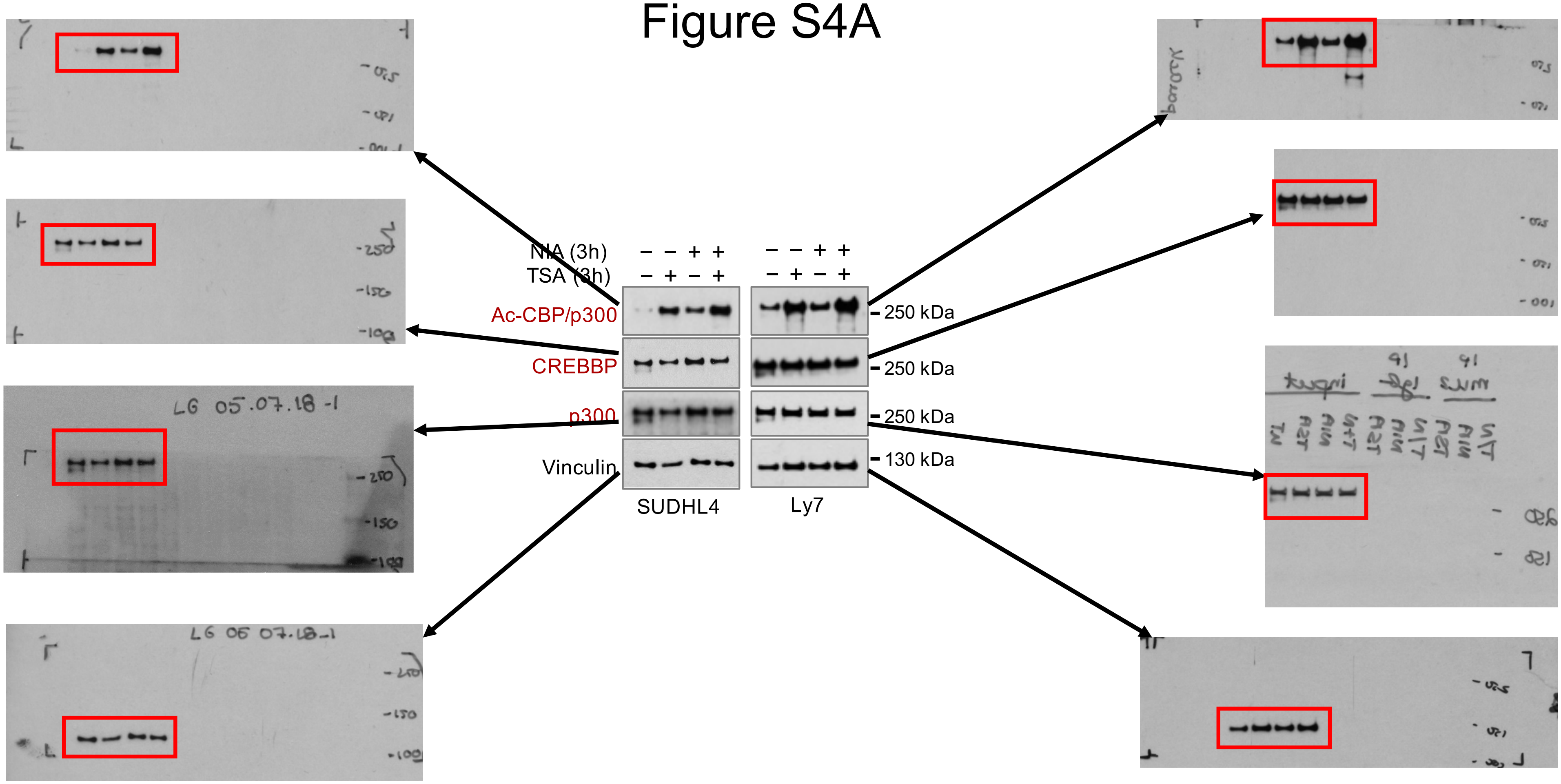

NIA (3h) – – + + – – + +
TSA (3h) – + – + – + – +
Ac-CBP/p300
CREBBP
p300
Vinculin
250 kDa
250 kDa
250 kDa
130 kDa
SUDHL4
Ly7
LG 05.07.18 -1

Figure S4C

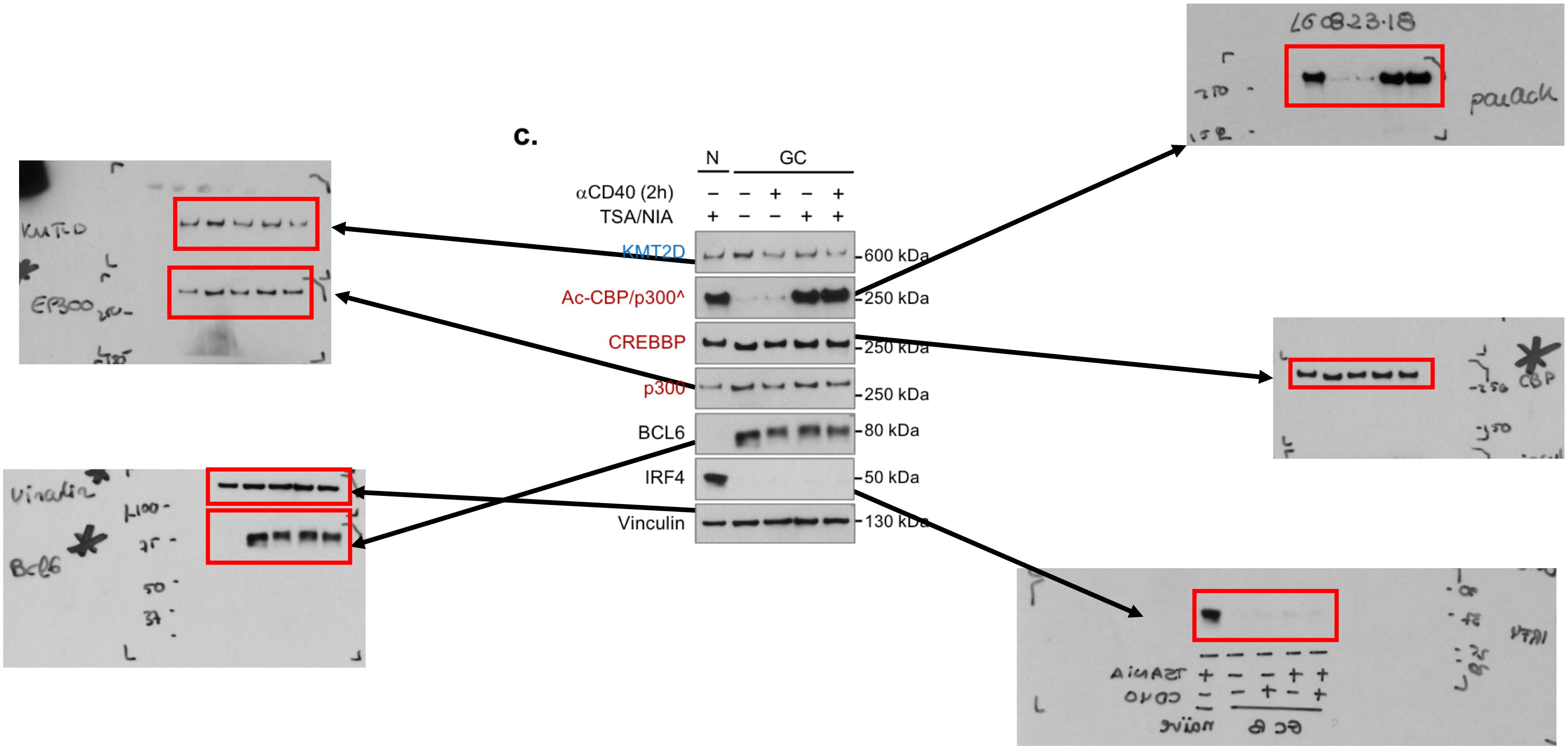
c.
N
GC
αCD40 (2h)
TSA/NIA
KMT2D
Ac-CBP/p300^
CREBBP
p300
BCL6
IRF4
Vinculin
600 kDa
250 kDa
250 kDa
250 kDa
80 kDa
50 kDa
130 kDa

Figure S6B

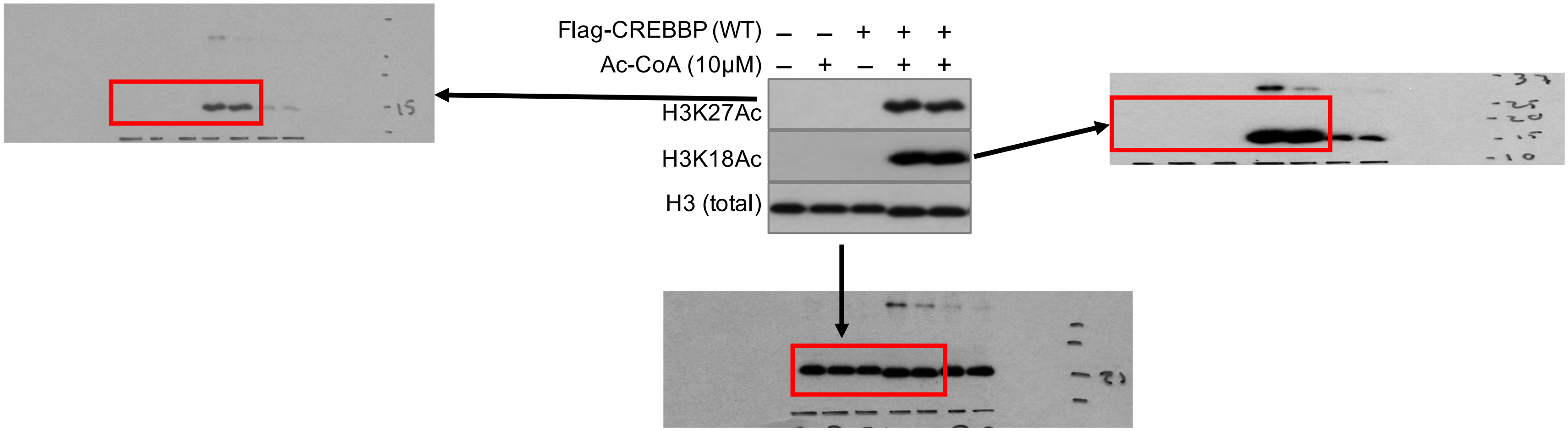

Figure S6C

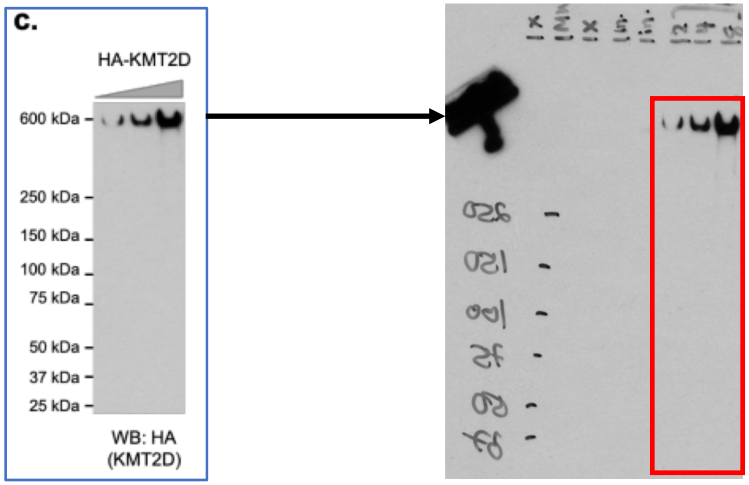
c.
HA-KMT2D
600 kDa
250 kDa
150 kDa
100 kDa
75 kDa
50 kDa
37 kDa
25 kDa
WB: HA
(KMT2D)
